# Deep learning radiomics based on contrast enhanced MRI for preoperatively predicting early recurrence in hepatocellular carcinoma after curative resection

**DOI:** 10.3389/fonc.2024.1446386

**Published:** 2024-11-08

**Authors:** Ying Zhao, Sen Wang, Yue Wang, Jun Li, Jinghong Liu, Yuhui Liu, Haitong Ji, Wenhan Su, Qinhe Zhang, Qingwei Song, Yu Yao, Ailian Liu

**Affiliations:** ^1^ Department of Radiology, The First Affiliated Hospital, Dalian Medical University, Dalian, China; ^2^ Chengdu Institute of Computer Application, Chinese Academy of Sciences, Chengdu, China; ^3^ School of Computer Science and Technology, University of Chinese Academy of Sciences, Beijing, China; ^4^ College of Medical Imaging, Dalian Medical University, Dalian, China; ^5^ Dalian Engineering Research Center for Artificial Intelligence in Medical Imaging, Dalian, China

**Keywords:** hepatocellular carcinoma, early recurrence, magnetic resonance imaging, deep learning, radiomics

## Abstract

**Purpose:**

To explore the role of deep learning (DL) and radiomics-based integrated approach based on contrast enhanced magnetic resonance imaging (CEMRI) for predicting early recurrence (ER) in hepatocellular carcinoma (HCC) patients after curative resection.

**Methods:**

Total 165 HCC patients (ER, *n* = 96 vs. non-early recurrence (NER), *n* = 69) were retrospectively collected and divided into a training cohort (*n* = 132) and a validation cohort (*n* = 33). From pretreatment CEMR images, a total of 3111 radiomics features were extracted, and radiomics models were constructed using five machine learning classifiers (logistic regression, support vector machine, k-nearest neighbor, extreme gradient Boosting, and multilayer perceptron). DL models were established via three variations of ResNet architecture. The clinical-radiological (CR), radiomics combined with clinical-radiological (RCR), and deep learning combined with RCR (DLRCR) models were constructed. Model discrimination, calibration, and clinical utilities were evaluated by receiver operating characteristic curve, calibration curve, and decision curve analysis, respectively. The best-performing model was compared with the widely used staging systems and preoperative prognostic indexes.

**Results:**

The RCR model (area under the curve (AUC): 0.841 and 0.811) and the optimal radiomics model (AUC: 0.839 and 0.804) achieved better performance than the CR model (AUC: 0.662 and 0.752) in the training and validation cohorts, respectively. The optimal DL model (AUC: 0.870 and 0.826) outperformed the radiomics model in the both cohorts. The DL, radiomics, and CR predictors (aspartate aminotransferase (AST) and tumor diameter) were combined to construct the DLRCR model. The DLRCR model presented the best performance over any model, yielding an AUC, an accuracy, a sensitivity, a specificity of 0.917, 0.886, 0.889, and 0.882 in the training cohort and of 0.844, 0.818, 0.800, and 0.846 in the validation cohort, respectively. The DLRCR model achieved better clinical utility compared to the clinical staging systems and prognostic indexes.

**Conclusion:**

Both radiomics and DL models derived from CEMRI can predict HCC recurrence, and DL and radiomics-based integrated approach can provide a more effective tool for the precise prediction of ER for HCC patients undergoing resection.

## Introduction

Hepatocellular carcinoma (HCC) is the most common primary hepatic malignancy and the third leading cause of cancer-related deaths worldwide (1). Although surgical resection has been considered as the first-line curative treatment for early-stage HCC patients with well-preserved liver function, the high recurrence rates after resection still remain a major hurdle, and 70% of the patients occur recurrence within 5 years (2). Patients with early recurrence (ER) within 2 years after operation are at high risk for poor prognosis (3), whose are potential candidates for clinical trials of adjuvant systemic therapies (4). Thus, the identification of patients with high recurrent risk at early stage is critical for prognostication surveillance and thus facilitating the implementation of individualized treatment.

Several identified predictors for HCC recurrence include various pathological markers related to tumor aggressiveness, such as tumor size, microvascular invasion, surgical margin, and Edmondson-Steiner grade, as reported in published studies (3, 5, 6). However, preoperative prediction of HCC recurrence risk remains difficult, and thus a noninvasive tool with adequate information about tumor characterization that enables to accurately estimate prognosis is needed. Magnetic resonance imaging (MRI) is the ideal technique for non-invasive diagnosis and surveillance of HCC due to its high soft tissue contrast and multiparametric imaging. Previous studies have reported that conventional radiological features including non-smooth tumor margin, incomplete/without tumor capsule, peritumoral enhancement, and hypointensity on hepatobiliary phase can predict clinical outcomes (7–9); however, these features interpreted by radiologists indicate high subjectivity, difficulty in quantification, and lack of metrics about tumor heterogeneity.

In recent years, radiomics has emerged as a promising tool to facilitate precision diagnosis and prognosis of HCC with increases in the scale of medical imaging data and development of artificial intelligence (AI) techniques. Radiomics can convert medical images into high-throughput and quantitative handcrafted features using computer algorithms to capture intratumoral pathophysiology and heterogeneity. Key radiomics features are selected and harnessed to construct robust and reproducible imaging markers for clinicians, from diagnosis assistance to therapeutic guidance (10, 11). Recent studies have utilized MRI-based radiomics containing intratumoral and/or peritumoral regions to predict HCC recurrence with promising predictive results (12, 13).

Furthermore, deep learning (DL), as the state-of-the-art machine learning (ML) technique in the field of AI, has been successfully applied in many pattern recognition tasks, which can bring revolutionary changes in health care (14). DL is a type of representation learning method allowing computational models that are composed of multiple processing layers to automatically learn representations of data by transforming the input information into multiple levels of abstractions (14, 15). It has been proven to be very good at discovering intricate structures in high-dimensional data and is therefore applicable to reveal complex relationships between multimodal images and challenging clinical questions with very little engineering by hand (15, 16). Previous studies of deep learning applied to pathological indicator classification and prognosis prediction in HCC have reported superior performance compared to those by conventional imaging modality or even better than radiomics (17, 18). Notely, a ML framework integrating handcrafted radiomics features with DL features has an emerging trend for achieving satisfying predictive performance in some specific clinical tasks (18, 19). Nevertheless, only a few studies (18, 20) have applied DL and radiomics-based integrated approach for HCC recurrence prediction, in which the traditional ML method was used for dimensionality reduction and fusion model construction.

The aim of the present study was to develop and validate radiomics and DL models based on preoperative multi-phase contrast enhanced magnetic resonance imaging (CEMRI) for predicting ER of HCC patients after curative resection. Furthermore, we evaluated the predictive capacity of the combined model incorporating DL, radiomics, and clinical-radiological features. We assumed that the proposed DL and radiomics-based integrated approach can improve recurrence prediction accuracy, thus creating a better risk stratification and enhancing the overall prognosis of HCC patients after surgical resection.

## Materials and methods

### Patients

**Figure 1:**
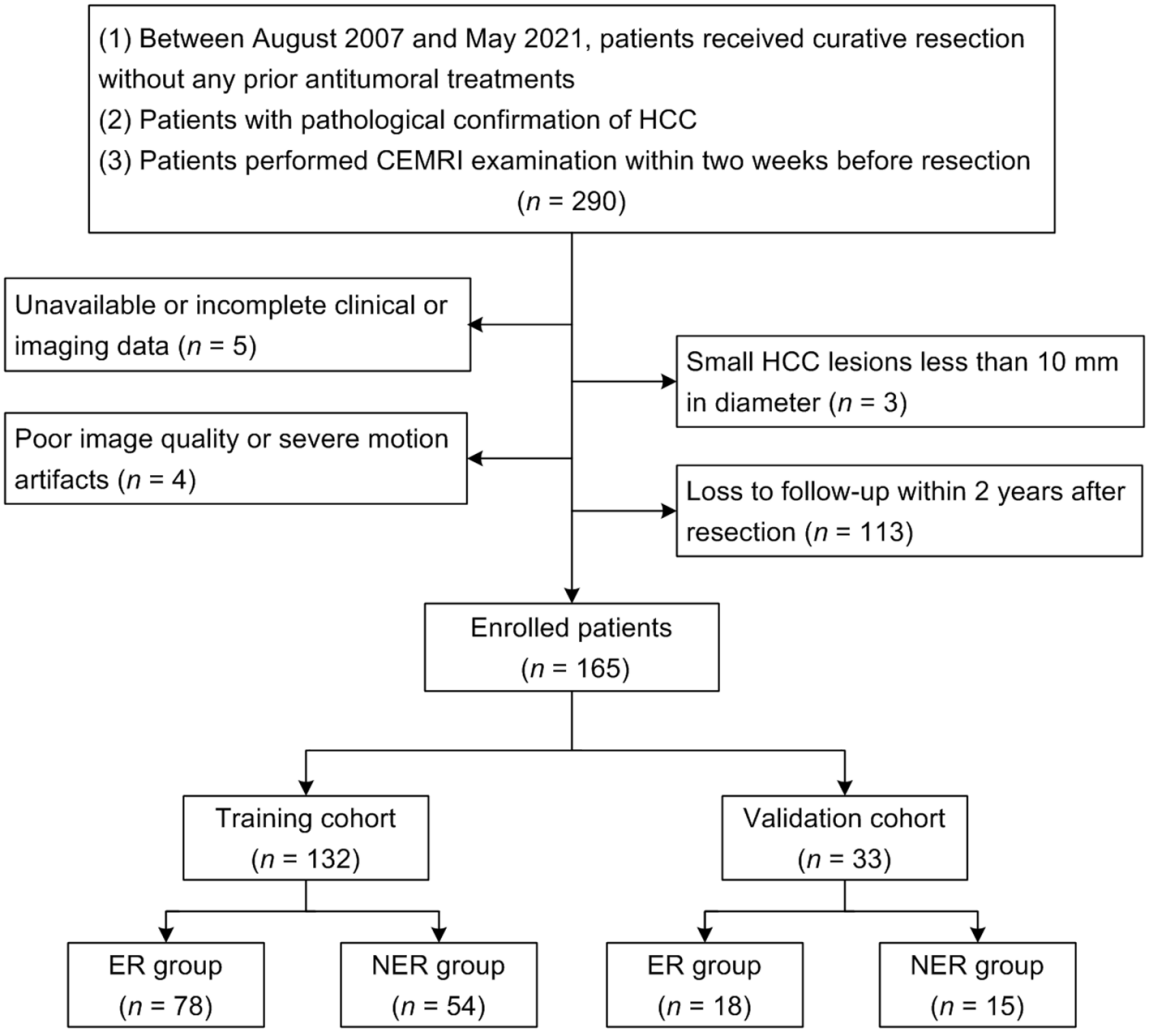
The flow chart of this study population.

This retrospective study was approved and the requirement for the informed consent was waived by the Institutional Review Board of our hospital (approval number: PJ-KS-KY-2022-180), and was carried out in accordance with the Declaration of Helsinki. Between August 2007 and May 2021, we retrospectively recruited 290 consecutive patients with pathologically confirmed HCC who performed preoperative abdominal MRI examination at our institution. The inclusion criteria were as follows: (1) patients received curative (R0) resection without any prior antitumoral treatments; (2) patients with pathological confirmation of HCC; (3) patients performed CEMRI examination within two weeks before resection. The exclusion criteria were as follows: (1) unavailable or incomplete clinical or imaging data (*n* = 5); (2) small HCC lesions less than 10 mm in diameter (*n* = 3); (3) poor image quality or severe motion artifacts (*n* = 4); (4) loss to follow-up within 2 years after resection (*n* = 113). The flow chart of this study population is shown in **Figure 1**. Ultimately, a total of 165 HCC patients were recruited in the study. The patients were randomly divided at a ratio of 8:2 into the training and validation cohorts.

Baseline clinical characteristics, including age, gender, history of hepatitis B or C, alpha-fetoprotein (AFP), alanine aminotransferase (ALT), aspartate aminotransferase (AST), γ-glutamyltranspeptadase (GGT), total bilirubin (TBIL), albumin (ALB), and Child-Pugh class, were retrospectively collected.

### MRI acquisition

MRI was performed using a 1.5 T or 3.0 T MR scanner (Signa, HDXT, GE Healthcare, USA) with an eight-channel phased array body coil. MRI protocols included in- and opposed-phase fast-spoiled gradient-recalled echo T1-weighted imaging (T1WI), fat-suppressed fast spin-echo T2-weighted imaging (T2WI), and contrast enhanced imaging with fat-suppressed T1-weighted fast-spoiled gradient-recalled echo sequence. The contrast enhanced images consisted of arterial phase (AP), portal venous phase (PVP), and delayed phase (DP) images, which were obtained at 40 s, 70 s, and 90 s, respectively. Gd-diethylenetriamine pentaacetic acid (Gd-DTPA) (Bayer Schering Pharma AG, Germany) was injected at a patient weight-dependent dose of 0.1 mmol/kg and an injection rate of 2.5 mL/s through a median cubital vein. Of the 165 HCC patients described above, 74 patients were examined with a 1.5 T MR system, and the other 91 patients with a 3.0 T MR system. Detailed parameters of imaging acquisition protocols are listed in **Supplementary Material S1**.

### Evaluation of MRI features

All MR images were retrospectively assessed by two radiologists with 10 (radiologist 1, Y.Z.) and 5 (radiologist 2, Y.W.) years of experience in abdominal MRI interpretation, who were blinded to clinical, pathological, and follow-up information. If there was any disagreement between radiologists during the imaging analysis, the images were evaluated by another senior radiologist of 20 (radiologist 3, J.H.L.) years of experience in abdominal MRI.

MRI features included the following: (1) tumor diameter (the maximum axial diameter including the capsule measured on PVP images) (21); (2) tumor number (unifocal or multifocal); (3) tumor margin (smooth or non-smooth); (4) intratumor necrosis (hypointensity on T1WI, hyperintensity on T2WI, and no enhancement of part of the tumor on all enhanced phases); (5) intratumor hemorrhage (heterogeneous hyperintensity on T1WI and hypointensity on T2WI); (6) tumor encapsulation (a peripheral rim of uniform and smooth hyperenhancement on PVP or DP images) (22); (7) arterial peritumoral enhancement (a zone of irregular and patchy hyperenhancement surrounding the tumor on AP images, becoming isointensity compared with normal liver parenchyma on DP images) (23); (8) radiological cirrhosis (surface irregularity and nodularity, shrunken of liver, widening of fissures, accompanied by ascites or signs of portal hypertension).

### Follow up

All patients were regularly followed up once every 3 months for 2 years after curative resection. Serum AFP level, liver function tests, and imaging examinations (included contrast enhanced computed tomography (CECT) or CEMRI) were conducted to monitor recurrence of HCC. The censored follow-up date was May 2023. Early recurrence was defined as new intrahepatic lesions and/or extrahepatic metastasis within 2 years after resection confirmed by typical imaging features or histopathology.

### Image segmentation and preprocessing

Preoperative MR images of AP, PVP, and DP were exported as digital imaging data and communications in medicine (DICOM) format. The images in DICOM format were converted to NIFTI format. All images were resampled to the same voxel size of 1 × 1 × 1 mm via linear interpolation algorithm to standardize the voxel spacing. Intensity normalization of images was performed to correct the scanner effect. Three dimensional segmentation of the whole tumor was performed on each phase using open source software ITK-SNAP (version 3.6.0, http://www.itksnap.org/). The volume of interests (VOIs) of all patients were manually delineated slice-by-slice along the visible borders of the tumor by radiologist 1 (Y.Z.). In terms of multifocal HCCs, the largest nodule was selected as the delineated lesion. Thirty tumors were randomly selected and then repeatedly segmented by two abdominal radiologists (radiologists 1 and 2, Y.Z. and Y.W.) independently to evaluate the intra- and inter-observer reproducibility of the radiomics features.

### Radiomics feature extraction

A total of 1037 radiomics features were extracted from each enhanced phase using Pyradiomics package implemented in Python (version 3.7.11, https://www.python.org/). Radiomics features were comprised of the following five categories: (1) histogram features (*n* = 18); (2) shape features (*n* = 14); (3) texture features (*n* = 75, including gray level co-occurrence matrix (GLCM), gray level run length matrix (GLRLM), neighborhood gray tone difference matrix (NGTDM), gray level dependence matrix (GLDM), and gray level size zone matrix (GLSZM)); (4) wavelet features (*n* = 186); (5) Laplacian of Gaussian features (*n* = 744). The extracted radiomics features were in accordance with feature definitions described by the image biomarker standardization initiative (IBSI) reporting guidelines (24). Detailed descriptions of these radiomics features are provided in **Supplementary Material S2**. Next, values of radiomics features were standardized using the z-score normalization based on the mean and standard deviation values from the training cohort to eliminate the differences in the value scales of the radiomics features (19).

### Feature selection and radiomics model construction

**Figure 2:**
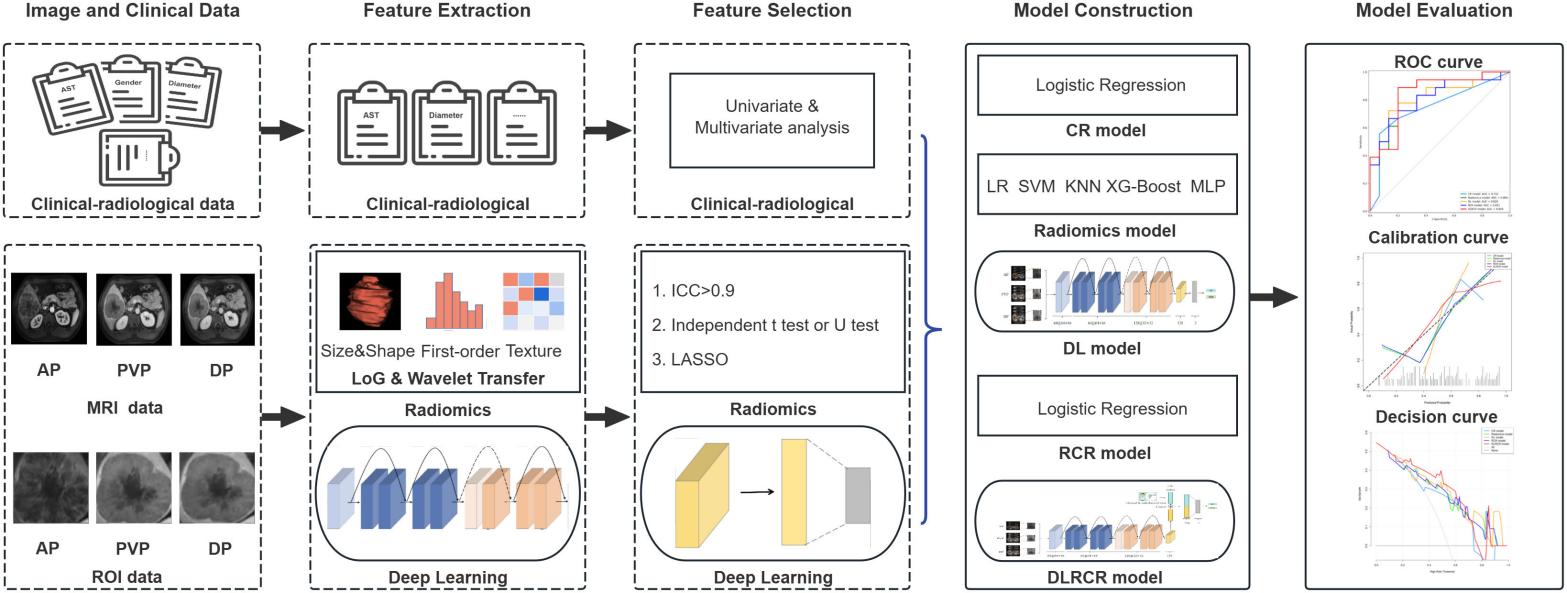
The workflow of radiomics and deep learning (DL) analysis in the current study. The volume of interests (VOIs) from contrast enhanced MR images in three phases [arterial phase (AP), portal venous phase (PVP), and delayed phase (DP)] were extracted for feature development in both radiomics and DL models. The clinical and radiological data were collected for clinical-radiological (CR) model construction. Following feature selection, chosen radiomics features were merged with CR risk factors to build an integrated radiomics combined with clinical-radiological (RCR) model. Furthermore, DL features were combined with both CR and radiomics features to develop a comprehensive deep learning combined with RCR (DLRCR) model. The discrimination, calibration, and clinical utilities were evaluated by receiver operating characteristic curve, calibration curve, and decision curve analysis, respectively.

**Figure 2** shows the workflow of radiomics and DL analysis. We devised a three-step strategy for dimensionality reduction and robust feature selection. First, the intraclass correlation coefficients (ICCs) were used to assess the intra- and inter-observer reproducibility of radiomics features, and features with ICC > 0.90 (excellent stability) were selected. Second, the independent sample *t* test or Mann-Whitney *U* test was conducted to select the features that were statistically different between ER and non-early recurrence (NER) groups. The *P* value threshold for the significant features was set at 0.05. Finally, the least absolute shrinkage and selection operator (LASSO) algorithm, with penalty parameter tuning conducted by 5-fold cross-validation, was then utilized to identify the most top-ranked features for predicting HCC recurrence. Five commonly used ML classifiers, including logistic regression (LR), support vector machine (SVM), k-nearest neighbor (KNN), extreme gradient Boosting (XG-Boost), and multilayer perceptron (MLP), were used to build radiomics models.

### Deep learning model construction

For DL analysis, the PVP and DP images were registered to AP images respectively using QuickRigid registration of the Antspy (https://github.com/ANTsX/ANTsPy). Three consecutive slices with the maximum cross-sectional tumor area were selected, and the tumor regions were cropped and resized into 256 × 256 pixels. Considering that a large amount of training data could improve the performance of the model, we adopted data augmentation strategies to increase the number of ROIs, including rotation, scaling, flipping, and shifting. Since ResNet architecture has showed promising performance in multiple computer vision tasks (25), we chose two variations of ResNet architecture with different numbers of layers, including ResNet-18 and ResNet-34, as our basic architecture for model training. Because the parameters of the two variations are excessive and an overfitting error easily occurs in the small-scale liver tumor image datasets, we optimized the original ResNet-18 architecture by reducing the network’s depth to ResNet-10.

**Figure 3:**
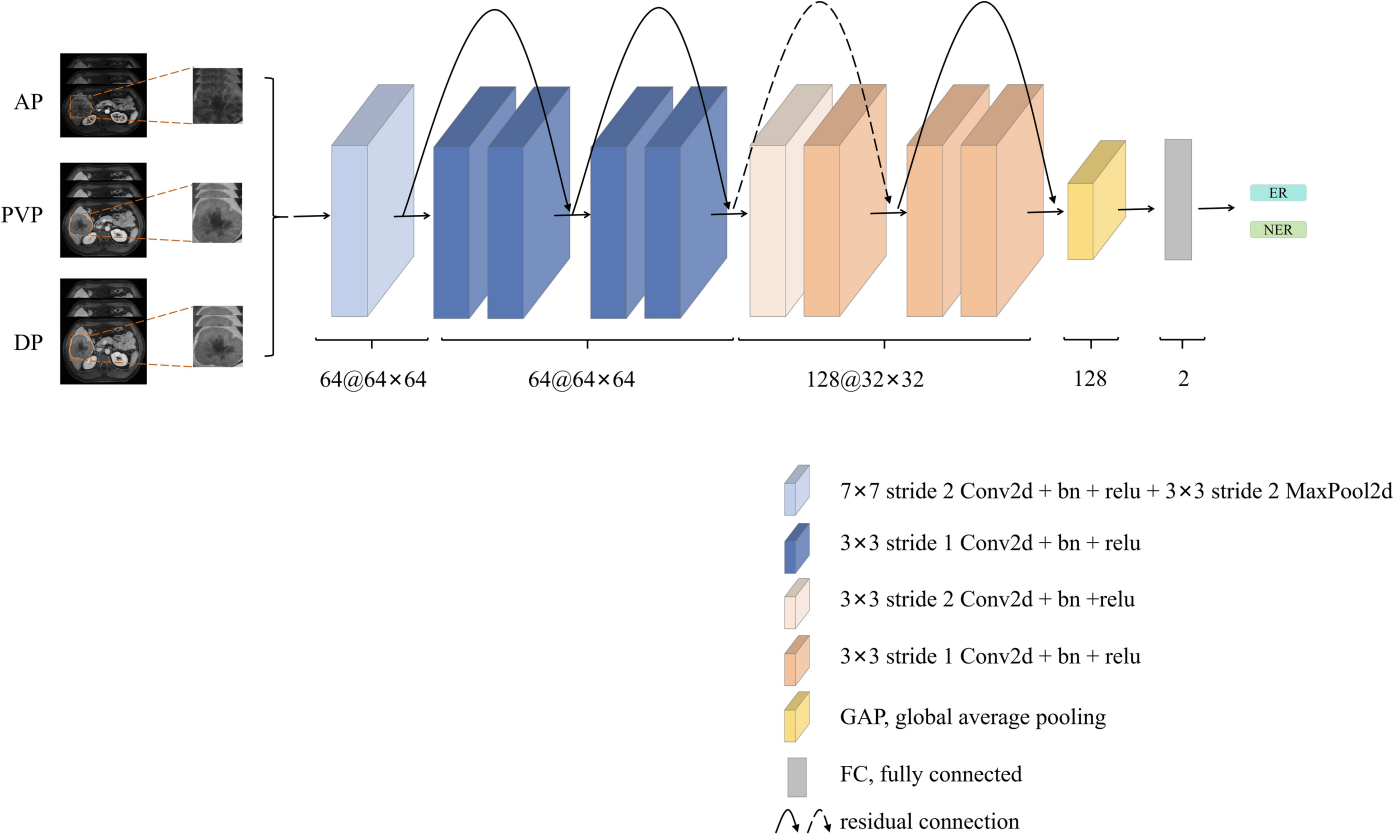
The network architecture of the deep learning (DL) model. The architecture involved concatenating three phase contrast enhanced MR images [arterial phase (AP), portal venous phase (PVP), and delayed phase (DP)], which were then fed into the ResNet-10. Subsequent to convolutional layers feature extraction, early recurrence (ER) prediction was performed through the fully connected layer. This architecture utilized the combination of multi-phase information and ResNet-10’s convolutional capabilities to achieve accurate ER prediction.

The detailed network architecture of the CNN is shown in **Figure 3**. The first part of the CNN included a 64 7×7 convolution layer, a normalization module, and a max pooling layer. After going through all these layers, we obtained a feature map of the input ROI. The following structures of the CNN contained four 64 3×3 convolution bottleneck modules and four 128 3×3 convolution bottleneck modules, in which all the bottleneck modules were employed from the ResNet. The raw feature map of the ROI was sequentially processed by these bottleneck modules, enabling the acquisition of ROI features at distinct scales. Shallow features learn more detailed structure information, while deep features can express high-level semantic features. After the last convolution operation, feature maps extracted by the backbone network were converted into feature vectors through global average pooling. These feature vectors were then concatenated and fed into the fully connected layer for HCC recurrence prediction. Additionally, to better explore the important features or patterns the DL model identified, we employed Score-CAM method for visual interpretation (26). Unlike gradient-based methods, Score-CAM removes the reliance on gradients by determining the weight of each activation map based on its forward-passing score for the target class. The final results were obtained through a linear combination of these weights and the corresponding activation maps.

The training and testing processes were conducted on the platform of GeForce GTX3060 graphics processing unit (NVIDIA, Santa Clara, Calif). The proposed framework was implemented by the Python programming language (https://www.python.org/) on the open source deep learning framework MONAI in conjunction with PyTorch (version 1.9.0 https://pytorch.org). The cross-entropy loss function of the DL model was minimized by the gradient descent algorithm. The number of training iteration was 200, and the batch size was 64. To reduce the risk of overfitting, the technique of early stopping was adopted. The learning rate was initialized by 1e-4 with the decay value of 1e−4 and momentum of 0.9.

### Clinical-radiological and combined models construction

To identify clinical and radiological predictors associated with HCC recurrence, the univariate logistic regression was employed and factors with *P* value of < 0.05 were further included in the multivariate logistic regression analysis. A clinical-radiological (CR) model was established based on the chosen CR independent risk factors by using logistic regression.

The radiomics combined with clinical-radiological (RCR) model, which incorporated the selected radiomics features derived from the highest performance radiomics model with CR risk factors for predicting tumor recurrence, was established using the proposed best-performing ML classifier.

These radiomics features belong to the low-level features of the manual design, while ResNet extracts the high-level semantic features. The combination of high-level DL features with CR and radiomics features can better describe the characteristics of the tumor and improve the capability of HCC recurrence prediction. We identified the DL model with the best performance for recurrence prediction based on the AUC and confirmed the preferably ResNet architecture variation. The high-level DL features and CR and radiomics features were combined with the fully connected layer to conduct joint training via the best-performing ResNet architecture variation, and were further applied to construct deep learning combined with RCR (DLRCR) model. Detailed diagram depicting the proposed fusion network architecture is listed in **Supplementary Material S3**. The DLRCR model performance was compared with the widely used Barcelona Clinic Liver Cancer (BCLC) staging system (27) and Chinese National Liver Cancer (CNLC) staging system (28), preoperative Early Recurrence After Surgery for Liver Tumor (ERASL) model (29), and some inflammation-based prognostic indexes (neutrophil-to-lymphocyte ratio (NLR), platelet-to-lymphocyte ratio (PLR), and lymphocyte-to-monocyte ratio (LMR)) (30).

### Statistical analysis

R software (version 3.4.1, https://www.r-project.org/) and Python software (version 3.7.11, https://www.python.org/) were used for statistical analyses. Continuous variables among clinical-radiological characteristics were compared using the independent sample *t* test or Mann-Whitney *U* test, and categorical variables were analyzed using the chi-squared test or Fisher’s exact test. The inter-observer consistency of radiological features was assessed using Kappa test (kappa value determination: κ > 0.80, excellent; 0.60 < κ ≤ 0.80, substantial; 0.40 < κ ≤ 0.60, moderate; κ ≤ 0.40, poor). To evaluate the performance of different models, predictive accuracy, sensitivity, and specificity were measured using receiver operating characteristic (ROC) curve and the area under the curve (AUC) was calculated. Comparisons between the AUCs of different predictive models were performed using the Delong’s test. We also performed stratified analysis on the subgroups of MRI scanner of the optimal radiomics model. We used ROC curve and AUC to evaluate model performance on the subpopulations. Model fit was assessed via calibration curves. The clinical utility of the models was evaluated using decision curve analysis (DCA). All statistical tests were two-sided, and *P* < 0.05 was considered statistically significant.

## Results

### Baseline characteristics

The final study cohort consisted of 165 patients (male: 138; median age, 59 years; range, 31-83 years) and was divided into the training cohort (*n* = 132) and the validation cohort (*n* = 33). Among the 165 patients, 96 (58.18%) patients were diagnosed with ER within 2 years, and 69 (41.82%) patients did not have ER. There was not significantly different in the ER rate between the training and validation cohorts (59.09% vs. 54.55%, *P* = 0.782). The median time to recurrence for all patients with ER was 7 months (range, 1-24 months). No significant differences of clinical-radiological characteristics were found between the training and validation cohorts (*P* = 0.081-1.000). Clinical-radiological data are summarized in **Table 1**.

**Table 1:** Patient clinical-radiological characteristics.

Characteristics	Training cohort (*n* = 132)			Validation cohort (*n* = 33)			*P* value
	ER group (*n* = 78)	NER group (*n* = 54)	*P* value	ER group (*n* = 18)	NER group (*n* = 15)	*P* value	
Gender (*n*, [%])			0.355			1.000	0.958
Male	68 (87.2)	43 (79.6)		15 (83.3)	12 (80.0)		
Female	10 (12.8)	11 (20.4)		3 (16.7)	3 (20.0)		
Age (years, mean ± SD)	58.35 ± 10.59	58.38 ± 10.60	0.604	57.81 ± 10.84	56.81 ± 10.26	0.758	0.642
History of hepatitis B or C (*n*, [%])			0.733			1.000	0.199
Positive	61 (78.2)	40 (74.1)		11 (61.1)	10 (66.7)		
Negative	17 (21.8)	14 (25.9)		7 (38.9)	5 (33.3)		
AFP (IU/ml) (*n*, [%])			0.046			0.607	0.397
≤ 400	57 (73.1)	48 (88.9)		15 (83.3)	14 (93.3)		
> 400	21 (26.9)	6 (11.1)		3 (16.7)	1 (6.67)		
ALT (U/L) (*n*, [%])			0.234			1.000	1.000
≤ 50	52 (66.7)	42 (77.8)		13 (72.2)	11 (73.3)		
> 50	26 (33.3)	12 (22.2)		5 (27.8)	4 (26.7)		
AST (U/L) (*n*, [%])			0.016			1.000	0.128
≤ 40	42 (53.8)	41 (75.9)		14 (77.8)	12 (80.0)		
> 40	36 (46.2)	13 (24.1)		4 (22.2)	3 (20.0)		
GGT (U/L) (*n*, [%])			0.084			0.923	0.785
≤ 60	36 (46.2)	34 (63.0)		11 (61.1)	8 (53.3)		
> 60	42 (53.8)	20 (37.0)		7 (38.9)	7 (46.7)		
TBIL (umol/L) (*n*, [%])			0.424			0.108	0.081
≤ 19	53 (67.9)	41 (75.9)		14 (77.8)	15 (100)		
> 19	25 (32.1)	13 (24.1)		4 (22.2)	0 (0.00)		
ALB (g/L) (*n*, [%])			0.624			0.266	1.000
< 40	29 (37.2)	17 (31.5)		4 (22.2)	7 (46.7)		
≥ 40	49 (62.8)	37 (68.5)		14 (77.8)	8 (53.3)		
Child-Pugh class (*n*, [%])			1.000			—	0.127
A	71 (91.0)	49 (90.7)		18 (100)	15 (100)		
B	7 (9.0)	5 (9.3)		0 (0.00)	0 (0.00)		
Tumor diameter (*n*, [%])			0.024			0.009	0.899
≤ 5 cm	48 (61.5)	44 (81.5)		8 (44.4)	14 (93.3)		
> 5 cm	30 (38.5)	10 (18.5)		10 (55.6)	1 (6.7)		
Tumor number (*n*, [%])			0.442			0.346	0.776
Unifocal	66 (84.6)	49 (90.7)		14 (77.8)	14 (93.3)		
Multifocal	12 (15.4)	5 (9.3)		4 (22.2)	1 (6.7)		
Tumor margin (*n*, [%])			0.567			0.266	0.550
Smooth	44 (56.4)	34 (63.0)		10 (55.6)	12 (80.0)		
Non-smooth	34 (43.6)	20 (37.0)		8 (44.4)	3 (20.0)		
Intratumoral necrosis (*n*, [%])			0.523			1.000	0.399
Present	30 (38.5)	17 (31.5)		8 (44.4)	7 (46.7)		
Absent	48 (61.5)	37 (68.5)		10 (55.6)	8 (53.3)		
Intratumoral hemorrhage (*n*, [%])			0.258			0.064	0.573
Present	24 (30.8)	11 (20.4)		9 (50.0)	2 (13.3)		
Absent	54 (69.2)	43 (79.6)		9 (50.0)	13 (86.7)		
Tumor encapsulation (*n*, [%])			0.284			0.674	0.891
Present	56 (71.8)	44 (81.5)		15 (83.3)	11 (73.3)		
Absent	22 (28.2)	10 (18.5)		3 (16.7)	4 (26.7)		
Arterial peritumoral enhancement (*n*, [%])			0.424			0.447	0.525
Present	25 (32.1)	13 (24.1)		5 (27.8)	7 (46.7)		
Absent	53 (67.9)	41 (75.9)		13 (72.2)	8 (53.3)		
Radiological cirrhosis (*n*, [%])			0.731			1.000	0.133
Present	54 (69.2)	35 (64.8)		9 (50.0)	8 (53.3)		
Absent	24 (30.8)	19 (35.2)		9 (50.0)	7 (46.7)		

Data are shown as number of patients, with the percentage in parentheses. ER, early recurrence; NER, non-early recurrence; SD: standard deviation; AFP, alpha-fetoprotein; ALT, alanine aminotransferase; AST, aspartate aminotransferase; GGT,γ-glutamyltranspeptadase; TBIL, total bilirubin; ALB; albumin.

### Radiomics and DL models development and evaluation

A total of 3111 radiomics features for three phase CEMR images were extracted. After intra- and inter-observer reproducibility analysis, 2739 features had ICC ≥ 0.9 and were performed for further analysis. 961 features with significant differences that aided in predicting recurrence were then identified. LASSO algorithm ultimately allowed the selection of 34 features, which were input into five ML classifiers for radiomics models building. The selected radiomics features and the corresponding coefficients are presented in **Supplementary Material S4**.

The performance comparisons among five classifiers are shown in **Table 2**, and ROC curves are presented in **Supplementary Material S5**. The radiomics models showed moderate to good discrimination in the both cohorts (AUC: training cohort, 0.839 - 1.000; validation cohort, 0.626 - 0.804). It demonstrated that the LR classifier performed the best with the AUCs of 0.839 (95% confidence interval (CI), 0.772 - 0.906) and 0.804 (95%CI, 0.650 - 0.957) in the training and validation cohorts, respectively. There was no significant difference in the AUCs of LR classifier between the training and validation cohorts (*P* = 0.436), which indicated that the model showed non overfitting and high robust; while significant differences in the AUCs of SVM, KNN, XG-Boost, and MLP classifiers between the training and validation cohorts were found (*P* < 0.05). The accuracy, sensitivity, and specificity of the best-performing radiomics model (LR classifier) were 0.780, 0.885, and 0.630 in the training cohort, and 0.727, 0.833, and 0.600 in the validation cohort, respectively. The stratified analysis showed that the optimal radiomics model (LR classifier) was not influenced by MRI scanners with different magnetic field strength in the training cohort (1.5 T: AUC, 0.882 (95%CI, 0.796 - 0.968); 3.0 T: AUC, 0.834 (95%CI, 0.743 - 0.925), *P* = 0.457) and the validation cohort (1.5 T: AUC, 0.880 (95%CI, 0.696 - 1.000); 3.0 T: AUC, 0.762 (95%CI, 0.538 - 0.986), *P* = 0.430).

**Table 2:** Discrimination performance of different classifiers in the training and validation cohorts.

Model		AUC (95% CI)	Accuracy	Sensitivity	Specificity	*P* value
LR model	TC	0.839 (0.772 - 0.906)	0.780	0.885	0.630	0.436
	VC	0.804 (0.650 - 0.957)	0.727	0.833	0.600	
SVM model	TC	0.971 (0.935 - 1.000)	0.932	0.987	0.852	0.003
	VC	0.641 (0.439 - 0.842)	0.636	0.889	0.333	
KNN model	TC	0.874 (0.816 - 0.931)	0.803	0.821	0.778	0.031
	VC	0.637 (0.439 - 0.835)	0.515	0.500	0.533	
XG-Boost model	TC	0.934 (0.888 - 0.980)	0.871	0.846	0.907	0.028
	VC	0.722 (0.547 - 0.897)	0.667	0.556	0.800	
MLP model	TC	1.000 (1.000 - 1.000)	1.000	1.000	1.000	<0.001
	VC	0.626 (0.429 - 0.823)	0.606	0.667	0.533	

LR, logistic regression; SVM, support vector machine; KNN, k-nearest neighbor; XG-Boost, extreme gradient Boosting; MLP, multilayer perceptron; TC, training cohort; VC, validation cohort; AUC, area under the curve; CI, confidence interval.

**Figure 4:**
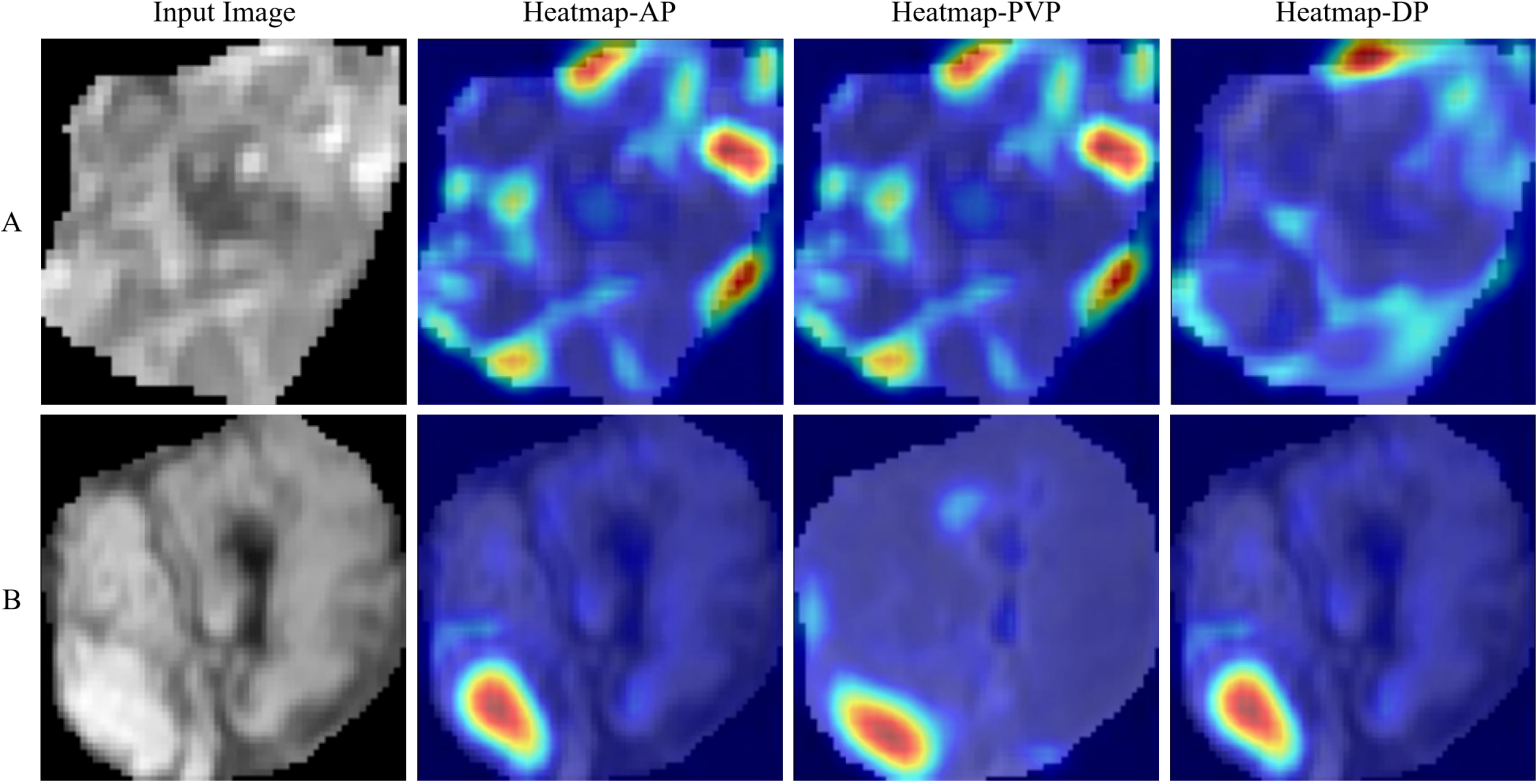
Visualization results for the early recurrence case **(A)** and non-early recurrence case **(B)**. Input images represent the cropped tumor regions fed into the model. Heatmaps are standard jet colormaps overlaid on the original input images, where red indicates areas of the highest relevance to the classification, followed by yellow, while green and blue regions indicate weaker predictive relevance. AP, PVP, and DP denote arterial phase, portal venous phase, and delayed phase, respectively.

Predictive performances and ROC curves of DL models are shown in **Supplementary Material S6**. The AUCs of ResNet-18 and ResNet-34 models were 0.700 (95%CI, 0.608 - 0.792) and 0.611 (95%CI, 0.511 - 0.712) in the training cohort, and 0.704 (95%CI, 0.517 - 0.890) and 0.619 (95%CI, 0.414 - 0.823) in the validation cohort, respectively. Compared with ResNet-18 and ResNet-34 models, the ResNet-10 model achieved the best performance with an AUC, an accuracy, a sensitivity, and a specificity of 0.870 (95%CI, 0.806 - 0.934), 0.803, 0.861, 0.733 in the training cohort, respectively, and an AUC, an accuracy, a sensitivity, and a specificity of 0.826 (95%CI, 0.682 - 0.970), 0.788, 0.824, and 0.750 in the validation cohort, respectively. The DL (ResNet-10) model obtained better performance than radiomics (LR) model (AUC: training, 0.870 vs. 0.839; validation, 0.826 vs. 0.804). In addition, we computed activation maps and visualized the AP, PVP, and DP images, where the darker the color in the activation map, the more significant the region’s importance. The highlighted regions in the map were primarily concentrated on the tumor margins. The visualization results of the DL model are showed in **Figure 4**.

### Clinical-radiological and combined models construction and evaluation

**Figure 5:**
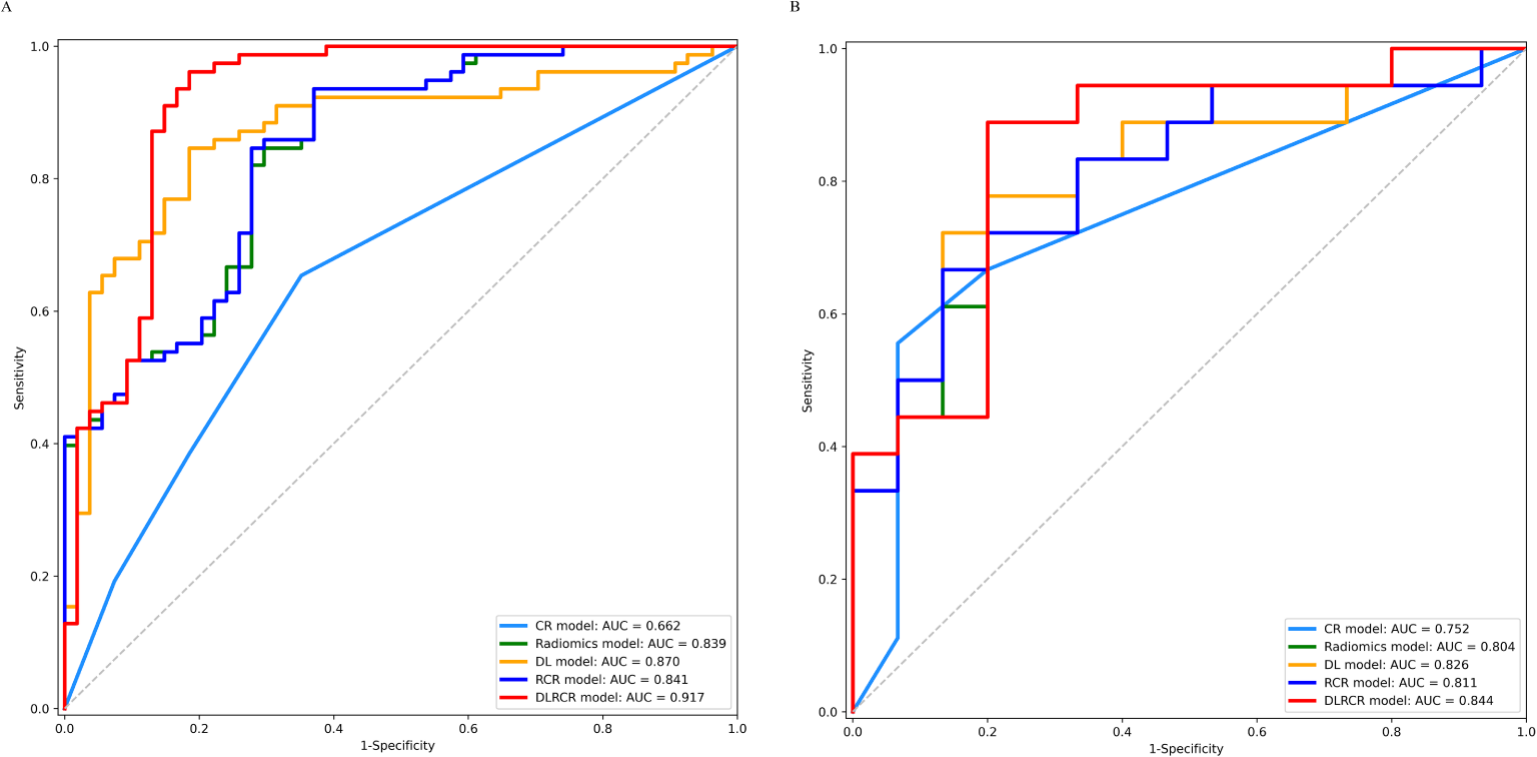
ROC curves for the clinical-radiological (CR) model, radiomics model, deep learning (DL) model, radiomics combined with clinical-radiological (RCR) model, and deep learning combined with RCR (DLRCR) model in the training cohort **(A)** and the validation cohort **(B)**.

Inter-observer agreements on the MR imaging features were excellent (kappa-value range: 0.834 to 1.000). Univariate and multivariate analyses of clinical-radiological characteristics for predicting tumor recurrence in the training cohort are shown in **Table 3**. The univariate analysis demonstrated that AFP, AST, and tumor diameter were significant CR factors for discriminating the ER and NER groups in the training cohort (all *P* < 0.05). The multivariate analysis showed that AST (OR = 2.490; 95% CI: 1.140 - 5.440; *P* = 0.020) and tumor diameter (OR = 2.510; 95% CI: 1.080 - 5.820; *P* = 0.030) were independent risk factors for predicting ER in HCC patients. The CR model was built based on the two risk factors, which achieved an AUC of 0.662 (95%CI, 0.574 - 0.749) in the training cohort and 0.752 (95%CI, 0.593 - 0.911) in the validation cohort, respectively (**Table 4**; **Figures 5A, B**).

**Table 3:** Univariate and multivariate analyses of clinical-radiological characteristics for predicting early recurrence in the training cohort.

Variables	Univariate analysis		Multivariate analysis	
	Odd ratio (95% CI)	*P* value	Odd ratio (95% CI)	*P* value
Gender	1.74 (0.68 - 4.44)	0.25	—	—
Age	1.01 (0.98 - 1.04)	0.65	—	—
History of hepatitis B or C	1.26 (0.56 - 2.83)	0.58	—	—
AFP	2.95 (1.10 - 7.89)	0.03	—	—
ALT	1.75 (0.79 - 3.88)	0.17	—	—
AST	2.70 (1.26 - 5.82)	0.01	2.49 (1.14 - 5.44)	0.02
GGT	1.98 (0.98 - 4.03)	0.06	—	—
T-BIL	1.49 (0.68 - 3.26)	0.32	—	—
ALB	0.78 (0.37 - 1.62)	0.50	—	—
Child-Pugh class	0.97 (0.29 - 3.22)	0.96	—	—
Tumor diameter	2.75 (1.21 - 6.27)	0.02	2.51 (1.08 - 5.82)	0.03
Tumor number	1.78 (0.59 - 5.39)	0.31	—	—
Tumour margin	1.31 (0.65 - 2.67)	0.45	—	—
Intratumoral necrosis	0.74 (0.35 - 1.53)	0.41	—	—
Intratumoral hemorrhage	0.58 (0.25 - 1.30)	0.19	—	—
Tumor encapsulation	1.73 (0.74 - 4.03)	0.20	—	—
Arterial peritumoral enhancement	0.67 (0.31 - 1.47)	0.32	—	—
Radiological cirrhosis	0.82 (0.39 - 1.71)	0.60	—	—

Variables with *P* < 0.05 in the univariate analysis were included in the multivariate logistic regression analysis. AFP, alpha-fetoprotein; ALT, alanine aminotransferase; AST, aspartate aminotransferase; GGT,γ-glutamyltranspeptadase; TBIL, total bilirubin; ALB; albumin.

**Table 4:** Discrimination performance of different models in the training and validation cohorts.

Model		AUC (95% CI)	Accuracy	Sensitivity	Specificity	*P* value
CR model	TC	0.662 (0.574 - 0.749)	0.652	0.654	0.648	0.334
	VC	0.752 (0.593 - 0.911)	0.727	0.667	0.800	
Radiomics model	TC	0.839 (0.772 - 0.906)	0.780	0.885	0.630	0.681
	VC	0.804 (0.650 - 0.957)	0.727	0.833	0.600	
DL model	TC	0.870 (0.806 - 0.934)	0.803	0.861	0.733	0.584
	VC	0.826 (0.682 - 0.970)	0.788	0.824	0.750	
RCR model	TC	0.841 (0.774 - 0.908)	0.773	0.872	0.630	0.722
	VC	0.811 (0.661 - 0.962)	0.727	0.833	0.600	
DLRCR model	TC	0.917 (0.963 - 0.972)	0.886	0.889	0.882	0.355
	VC	0.844 (0.702 - 0.987)	0.818	0.800	0.846	

CR, clinical-radiological; DL, deep learning; RCR, radiomics combined with clinical-radiological; DLRCR, deep learning combined with RCR; TC, training cohort; VC, validation cohort; AUC, area under the curve; CI, confidence interval.

**Figure 6:**
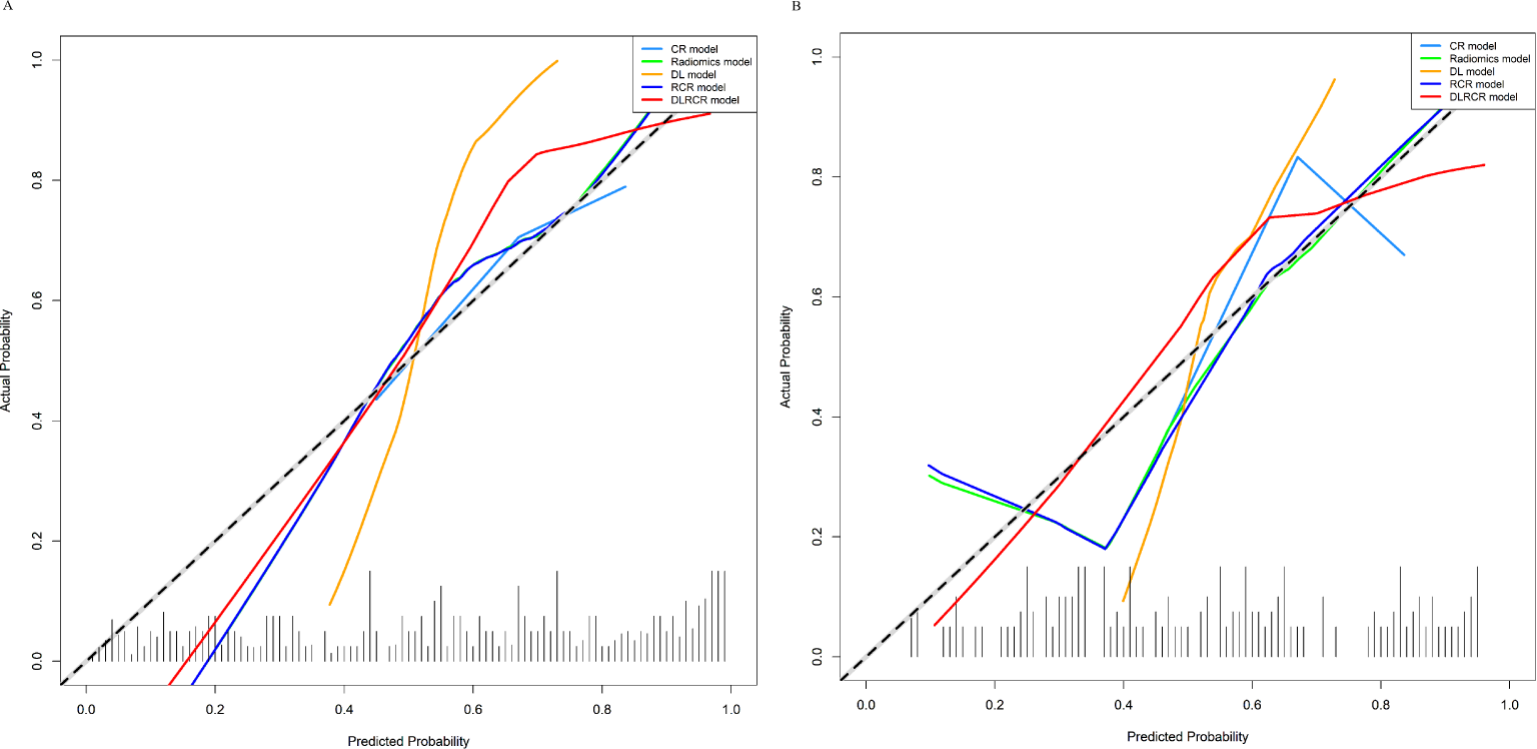
Calibration curves of various models in the training cohort **(A)** and the validation cohort **(B)**. The curves assess the models’ goodness-of-fit. The x-axis represents the predicted probability, and the y-axis represents the actual probability. The dashed line represents the ideal prediction by a perfect model. The solid line represents the predictive performance of the model. If the solid line is closer to the dashed line, it means a better goodness-of-fit.

**Figure 7:**
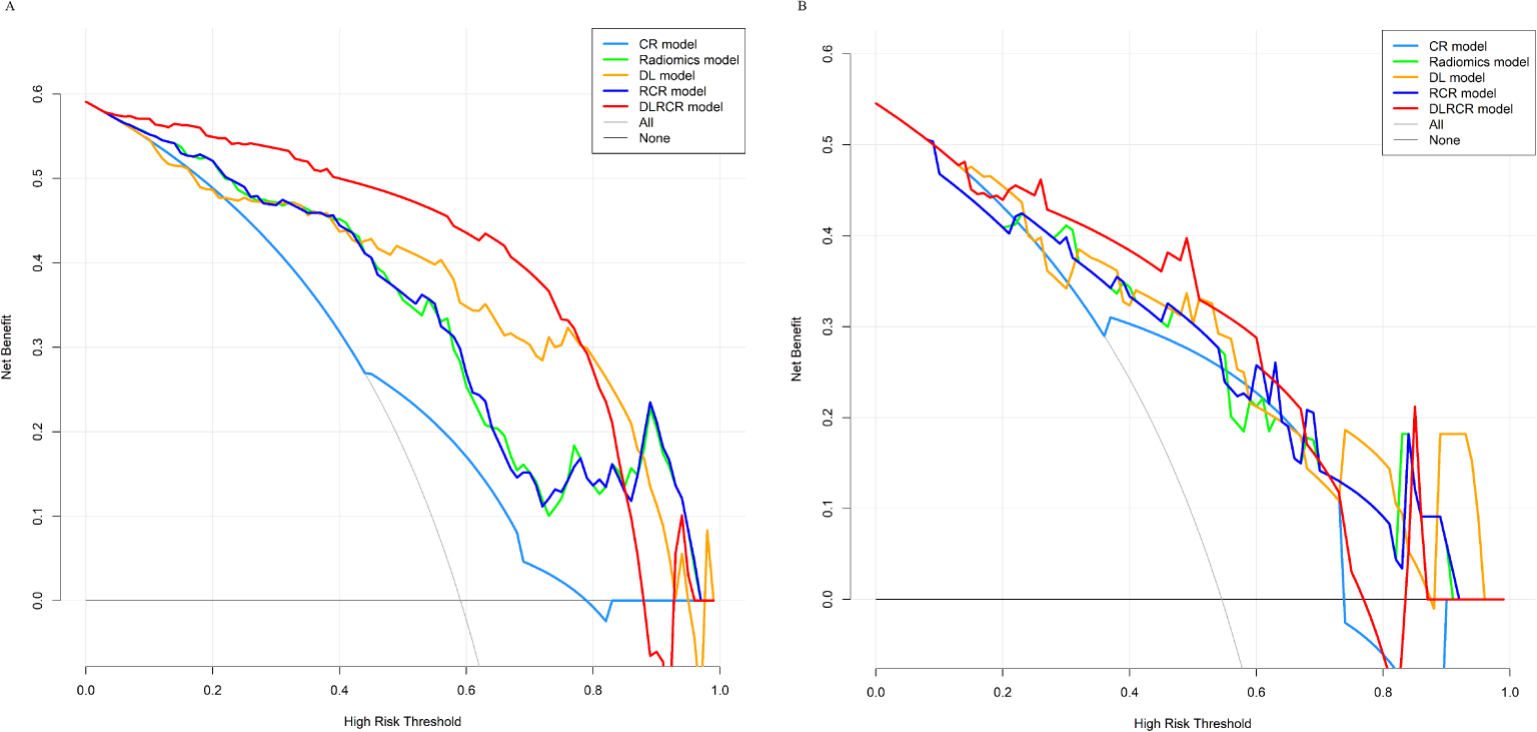
Decision curve analysis for different models in the training cohort **(A)** and the validation cohort **(B)**. The x-axis represents the threshold probability for high risk, and the y-axis denotes the net benefit. The DLRCR model obtained more net benefit compared with other models, treat-all strategy (gray line), and treat-none strategy (horizontal black line) across the majority range of threshold probabilities.

The RCR model was constructed integrating the remained radiomics features, AST, and tumor diameter using logistic regression classifier. Furthermore, the DL features, the chosen radiomics features, AST, and tumor diameter were input into the fully connected layer via ResNet-10 architecture to build the DLRCR model. The AUCs of RCR model were 0.841 (95%CI, 0.774 - 0.908) and 0.811 (95%CI, 0.661 - 0.962), which performed better than CR model (Delong’s test: training, *P* < 0.001; validation, *P* = 0.440). The DLRCR model outperformed the CR, radiomics, DL, and RCR models, yielding an AUC, an accuracy, a sensitivity, a specificity of 0.917 (95%CI, 0.963 - 0.972), 0.886, 0.889, and 0.882 in the training cohort and of 0.844 (95%CI, 0.702 - 0.987), 0.818, 0.800, and 0.846 in the validation cohort, respectively (**Table 4**; **Figures 5A, B**). The Delong’s test showed a significant difference of the AUCs between the DLRCR model and the CR model in the training cohort (*P* < 0.001), while there was no significant difference in the validation cohort (*P* = 0.232). Delong’s test of different predictive models in the both cohorts is shown in **Supplementary Material S7**. Calibration curves for the probability of ER demonstrated good model agreements between prediction and observation in the both cohorts (**Figures 6A, B**). DCA curves showed that the DLRCR model achieved more net benefit compared with other models for most of the threshold probabilities (**Figures 7A, B**).

In the training cohort, the DLRCR model demonstrated superior performance than BCLC system (0.565; 95%CI, 0.507 - 0.623), CNLC system (0.610; 95%CI, 0.529 - 0.692), preoperative ERASL model (0.550; 95%CI, 0.485 - 0.616), NLR (0.561; 95%CI, 0.476 - 0.646), PLR (0.544; 95%CI, 0.462- 0.626), and LMR (0.507; 95%CI, 0.422 - 0.592) (*P* < 0.05). In the validation cohort, the AUC of the DLRCR model was significantly higher than that of BCLC system (0.615; 95%CI, 0.513 - 0.717), preoperative ERASL model (0.572; 95%CI, 0.433 - 0.711), NLR (0.483; 95%CI, 0.307 - 0.660), PLR (0.467; 95%CI, 0.296 - 0.637), and LMR (0.489; 95%CI, 0.313 - 0.665) (*P* < 0.05), except for CNLC system (0.774; 95%CI, 0.635 - 0.914) (*P* = 0.492). Predictive performances and ROC curves of clinical prediction methods are provided in **Supplementary Material S8**.

## Discussion

In this study, we developed and validated radiomics and DL models based on multi-phase CEMR images using different ML classifiers and CNN architectures for preoperatively predicting early recurrence of HCC patients after curative resection. Furthermore, we proposed a novel DL and radiomics-based integrated approach for HCC recurrence prediction that combined DL, radiomics, and clinical-radiological data. Our study found that the optimal DL model improved the performance over the radiomics model. The DLRCR model exhibited superior predictive efficiency over any model in predicting HCC recurrence and better clinical utility compared to clinical prediction methods (BCLC system, CNLC system, preoperative ERASL model, and some inflammation-based prognostic indexes). This DL and radiomics-based integrated strategy can provide a promising tool for accurate prediction of recurrence, which may potentially guide individualized treatment and survival monitoring of HCC patients.

Early-stage HCC still has a high recurrence rate after resection. In our study, 58.18% of HCC patients occurred postoperative ER. Accurate preoperative prediction of ER is critical for risk stratification and appropriate therapeutic strategies adjustment, contributing to improve patients overall survival (OS). Currently, a number of studies have developed clinical model based on the independent risk factors to predict ER, which obtained the AUCs from 0.606 to 0.715 (13, 16, 21, 22). However, the estimated clinical metrics are limited and the predictive performance is still required to be improved. With the development of ML technology, a large amount of quantitative radiomics data have been used to construct more predictive models than those developed by clinical characteristics. In the present study, the multivariate analysis demonstrated that AST and tumor diameter were significant risk factors for ER prediction in patients with HCC, consistent with the results of other studies (3, 7, 12, 13). A recent study proved that the AST level > 40 U/L was independent predictor for poor recurrence-free survival (RFS) and OS (7). A multicenter study of 628 HCC patients demonstrated that the larger tumor diameter was closely associated with ER after liver resection for solitary HCC (3). We developed CEMRI-based radiomics models using five commonly used ML classifiers (LR, SVM, KNN, XG-Boost, and MLP) and evaluated its role in predicting ER. Our study showed that the LR classifier obtained better performance and robustness than the other four classifiers, which were consistent with previous studies (31–33). Logistic regression has advantages that it does not require a value with a normal distribution and can resist noisy interference and prevent overfitting using regularization (31). The radiomics model with LR classifier had a higher ability to predict ER than the clinical-radiological model (ΔAUC: training, 0.177; validation, 0.052). Furthermore, the combined model integrating clinical-radiological characteristics with radiomics features based on CEMRI was constructed and achieved improved performance compared with the clinical-radiological model (ΔAUC: training, 0.179; validation, 0.059). Nonetheless, the radiomics method relies on predefined feature engineering that the extraction and selection of “hand-engineered” features are still complicated, subjective, time-consuming, and lack of stable reproducibility, thus limiting their clinical applicability (34, 35).

The emerging DL approach has advantages in automatically learning and hierarchically organizing task-adaptive high-level image features with less manpower and time (36). It can achieve higher accuracy, reproducibility, and predictive performance compared to conventional radiomics method (37). Two recent studies utilizing DL algorithms based on preoperative CECT images showed moderate performances for HCC recurrence prediction with the AUCs of 0.723 and 0.730 in the validation cohort, respectively (38, 39). In contrast, the development of DL model using MR images maybe more promising due to the higher soft tissue contrast of MR imaging compared to CT. Kucukkaya et al. (40) conducted a MRI-based DL research on 120 HCC patients undergoing surgical resection, thermal ablation, or orthotopic liver transplantation for postoperative recurrence prediction within six different time frames (range 1 - 6 years), showing two-year recurrence and average AUCs of 0.750 and 0.760 in the test cohort, respectively. However, that study had limited sample size, lower predictive performance, and lacked of model comparisons of different CNN architectures. Gao et al. (18) reported that DL method based on multi-sequence MR images for predicting early recurrence of HCC, achieving the AUC and accuracy of 0.813 and 0.755 in the validation cohort, which were slightly lower than our DL model (AUC: 0.826; accuracy: 0.788). Compared to the above two studies (18, 40), our DL model are trained in an end-to-end fashion, which simplifies the process by eliminating intermediate steps and reducing potential sources of error thus improving predictive capacity. A recent study reported by Wang et al. (41) constructed a DL model based on Gd-EOB-DTPA-enhanced MRI using VGG-19 network for prediction of HCC early recurrence post-hepatectomy. Compared to the VGG-19 prediction model (41), our DL (ResNet-10) model has the following advantages: firstly, our model lies in its incorporation of residual connections, which allows it to achieve excellent performance with fewer parameters and effectively addresses the vanishing gradient problem during the training of deeper networks; secondly, we implemented a weighted approach for each class in the cross-entropy loss to address class imbalance in the network; finally, our model achieved better performance metrics in the validation cohort (AUC: 0.826 vs. 0.759; accuracy: 0.788 vs. 0.775).

In the current study, we established three DL models using different neural network layers of ResNet architecture (ResNet-10, ResNet-18, and ResNet-34), and the shallower ResNet-10 model obtained better ER predictive performance compared to the other two DL models (ΔAUC: training, 0.170 - 0.259; validation, 0.122 - 0.207). Overfitting is a problem in DL field that becomes more serious with the more superimposed neural network layers there are (42). Specifically, as the number of layers in a model increases, it can learn progressively more complex features and patterns. However, if the data is insufficient or lacks diversity, the model may finally learn irrelevant information (noise) that is specific to the training set, leading to a decline in performance in the validation or test set. In our study, we primarily focused on model simplification technique to tackle overfitting, which allowed us to achieve superior predictive performance by using a smaller number of neural network layers. And this improvement can be attributed to two key aspects: firstly, having fewer neural network layers results in a lower model complexity, which can help reduce the risk of overfitting, especially when training data is limited; secondly, shallow neural networks can effectively capture global patterns and key features within the data, particularly for tasks with relatively simple structures or lower dimensions, where deeper models often do not provide significant performance improvements. Some studies have reached similar results that shallow neural networks can achieve comparable or even superior performance to deeper networks for specific tasks (43, 44). Additionally, the visualization analysis of the DL model demonstrated that the highlighted regions in the heatmap were primarily concentrated on the tumor margins, indicating that the features in these areas are crucial in supporting the model’s decisions. This may be interpreted that the non-smooth tumor margin and incomplete/without tumor capsule are closely related to prognosis of HCC patients (7, 8).

Recently, the DL and radiomics-based integrated strategy incorporating interpretable radiomics features with high-level temporal and spatial DL features has been demonstrated as a state-of-the-art quantitative tool and can be used to predict tumor behavior and prognosis due to its higher predictive accuracy (18–20, 45). However, studies of using DL and radiomics-based model for predicting early recurrence in HCC patients after curative resection are scarce, and to the best of our knowledge, only two studies have been conducted recently (18, 20). In a recent study (20), the clinical & deep learning-based radiomics model and deep learning-based radiomics model based on AP and PVP images of preoperative CECT showed better distinguished performance than the radiological model for prediction of 3-year recurrence rate of HCC after resection (AUC: 0.831 vs. 0.796 vs. 0.732). Gao et al (18) have further constructed a DL and radiomics-based combined model based on multi-sequence MR images, which demonstrated superior discriminative ability than the DL or radiomics model alone for ER prediction (AUC: 0.840 vs. 0.813 vs. 0.780). However, that study ignored routine clinical and radiological information that were useful and interpretable for predicting recurrence. Our study found that the DLRCR model achieved improvements in the AUC, accuracy, sensitivity, and specificity for predicting ER compared to the DL model or the RCR model alone, indicating that the combination of high-level DL features along with radiomics and clinical-radiological data maximized the predictive performance of ER. Compared with the only MRI-related study reported by Gao et al. (18), our study may have the following advantages: firstly, our study contained routine clinical information and conventional radiological features, which could provide more information about tumor characteristics; secondly, our DLRCR model possessed higher accuracy (0.818 vs. 0.777), sensitivity (0.800 vs. 0.769), and specificity (0.846 vs. 0.779) than the combined model in the validation cohort in that study, although the AUC (0.844 vs. 0.840) was comparable; finally, as opposed to the traditional approach of employing machine learning for dimensionality reduction and model building, our primary objective is to construct a neural network through the integration of DL, radiomics, and clinical-radiological data, which may automatically learn complementary information from multimodal data and thereby enhancing model performance.

Our study has several limitations. Firstly, the potential selection bias may exist due to the retrospective nature of the study. Secondly, this was a single-center study with relatively small samples. Our study have employed various strategies such as data augmentation, regularization, early stopping, and model simplification techniques to avoid overfitting and improve predictive performance. A large-scale and multi-center dataset is required to validate reliability and robustness of prediction models and to provide a better generalization of our results in the future. Thirdly, the VOIs were outlined manually by radiologists, thus is time- and labor-consuming, and limiting the model usefulness of our study. Further research is considered to design a DL framework integrating HCC automatic segmentation and recurrence prediction. Fourthly, the DL method is regarded as “black box” and lacks interpretability that is a challenge to explain the correlation between relevant features and results. To interpret important features or patterns identified by the DL model, we performed visual interpretation using Score-CAM method. In the future, we will try to continuously optimize our DL network to improve its predictive performance and interpretability. Finally, the value of our method for improving long-term survival in patients with HCC remains unclear, we will further explore the capacity of MRI DL and radiomics-based integrated approach for predicting OS of HCC patients after surgical resection.

In conclusion, CEMRI-based radiomics and DL models performed well in predicting HCC early recurrence after curative resection. Importantly, a novel DL and radiomics-based combined model incorporating clinical-radiological, radiomics, and high level DL features was proposed as a more effective method for the prediction of early recurrence for HCC patients. The integrated approach has potential to refine the prognosis and guide individualized treatment strategies for patients with HCC.

## Data Availability

The raw data supporting the conclusions of this article will be made available by the authors, without undue reservation.
