# Supplementary material for: Deep learning radiomics based on contrast enhanced MRI for preoperatively predicting early recurrence in hepatocellular carcinoma after curative resection
Source: Front Oncol. 2024 Nov 8;14:1446386. doi: 10.3389/fonc.2024.1446386 (PMC11581961; doi:10.3389/fonc.2024.1446386)
Supplement: Supplementary file 1 [file DataSheet1.pdf]

Supplementary Data

S1. Magnetic resonance imaging sequences and parameters

S2. Detailed name and description of radiomics features

2.1 Histogram features

2.2 Shape features

2.3 Gray level co-occurrence matrix (GLCM) features

2.4 Gray level run length matrix (GLRLM) features

2.5 Neighbouring Gray Tone Difference Matrix (NGTDM) features

2.6 Gray level dependence matrix (GLDM) features

2.7 Gray Level Size Zone Matrix (GLSZM) features

2.8 Wavelet features

2.9 Laplacian of Gaussian (LOG) features

S3. Detailed diagram depicting the fusion network architecture

S4. The selected radiomics features and the corresponding coefficients

S5. ROC curves of five machine learning classifiers

S6. Predictive performances and ROC curves of DL models

S7. Delong's test of different predictive models in the training and validation cohorts

S8. Predictive performances and ROC curves of clinical prediction methods

S9. References

S1. Magnetic resonance imaging sequences and parameters

Acquisition sequences	Field strength	TR (ms)	TE (ms)	FOV (mm^2)	Matrix size	NEX	Section thickness (mm)	Fat suppression
T1-weighted IP and OP imaging	1.5T	5.8	4.2 and 2.1	400 × 360	256 × 170	0.70	5	No
	3.0T	4	2.5 and 1.2	400 × 360	288 × 224	0.69	5	No
T2-weighted spin-echo	1.5T	6667	92.6	440 × 330	256 × 256	2.00	6.5	Yes
	3.0T	7059	96.1	440 × 396	288 × 224	2.00	6	Yes
Contrast-enhanced imaging	1.5T	5.8	3.1	400 × 360	256 × 170	0.70	5	Yes
	3.0T	4	1.8	420 × 378	270 × 224	0.70	5	Yes

IP, in-phase; OP, opposed-phase; TR, repetition time; TE, echo time; FOV, field of view; NEX, number of excitation.

S2. Detailed name and description of radiomics features

2.1 Histogram features

First-order statistics describe the distribution of voxel intensities within the image region defined by the mask through commonly used and basic metrics.

Let X be a set of voxels included in the ROI N_p. $P(i)$ be the first order histogram with discrete intensity levels, where is the number of non-zero bins, equally spaced from 0 with a width defined in the parameter. $p(i)$ be the normalized first order histogram and equal to $\frac{P(i)}{N_p}$.

1) Energy: Energy is a measure of the magnitude of voxel values in an image. A larger values implies a greater sum of the squares of these values.

$$energy = \sum_{i=1}^{N_p} (\mathbf{X}(i) + c)^2$$

2) Total Energy: Total Energy is the value of Energy feature scaled by the volume of the voxel in cubic mm.

$$total\ energy = V_{voxel} \sum_{i=1}^{N_p} (\mathbf{X}(i) + c)^2$$

3) Entropy: Entropy specifies the uncertainty/randomness in the image values. It measures the average amount of information required to encode the image values.

$$entropy = -\sum_{i=1}^{N_g} p(i) \log_2 \big(p(i) + \epsilon\big)$$

4) Minimum:

$$minimum = \min(\mathbf{X})$$

5) 10th percentile: The 10th percentile of X.

6) 90th percentile: The 90th percentile of X.

7) **Maximum:** The maximum gray level intensity within the ROI.

$$maximum = \max(\mathbf{X})$$

8) **Mean:** The average gray level intensity within the ROI.

$$mean = \frac{1}{N_p}\sum_{i=1}^{N_p}\mathbf{X}(i)$$

9) **Median:** The median gray level intensity within the ROI.

10) **Interquartile Range:** Here P_{25} and P_{75} are the 25th and 75th percentile of the image array, respectively.

$$interquartile\ range = \mathbf{P}_{75} - \mathbf{P}_{25}$$

11) **Range:** The range of gray values in the ROI.

$$range = \max(\mathbf{X}) - \min(\mathbf{X})$$

12) **Mean Absolute Deviation (MAD):** Mean Absolute Deviation is the mean distance of all intensity values from the Mean Value of the image array.

$$MAD = \frac{1}{N_p}\sum_{i=1}^{N_p}|\mathbf{X}(i) - \bar{X}|$$

13) **Robust Mean Absolute Deviation (rMAD):** Robust Mean Absolute Deviation is the mean distance of all intensity values from the Mean Value calculated on the subset of image array with gray levels in between, or equal to the 10th and 90th percentile.

$$rMAD = \frac{1}{N_{10-90}}\sum_{i=1}^{N_{10-90}}|\mathbf{X}_{10-90}(i) - \bar{X}_{10-90}|$$

14) **Root Mean Squared (RMS):** RMS is the square-root of the mean of all the squared intensity values.

$$RMS = \sqrt{\frac{1}{N_p}\sum_{i=1}^{N_p}(\mathbf{X}(i) + c)^2}$$

15) Skewness: Skewness measures the asymmetry of the distribution of values about the Mean value. Depending on where the tail is elongated and the mass of the distribution is concentrated, this value can be positive or negative.

$$skewness = \frac{\mu_3}{\sigma^3} = \frac{\frac{1}{N_p}\sum_{i=1}^{N_p}(\mathbf{X}(i) - \bar{X})^3}{\left(\sqrt{\frac{1}{N_p}\sum_{i=1}^{N_p}(\mathbf{X}(i) - \bar{X})^2}\right)^3}$$

Where is the $3\mu 3^{rd}$ central moment.

16) Kurtosis: Kurtosis is a measure of the ‘peakedness’ of the distribution of values in the image ROI.

$$kurtosis = \frac{\mu_4}{\sigma^4} = \frac{\frac{1}{N_p}\sum_{i=1}^{N_p}(\mathbf{X}(i) - \bar{X})^4}{\left(\frac{1}{N_p}\sum_{i=1}^{N_p}(\mathbf{X}(i) - \bar{X})^2\right)^2}$$

Where is the $4\mu 4^{th}$ central moment.

17) Variance: Variance is the the mean of the squared distances of each intensity value from the Mean value.

$$variance = \frac{1}{N_p}\sum_{i=1}^{N_p}(\mathbf{X}(i) - \bar{X})^2$$

18) Uniformity: Uniformity is a measure of the sum of the squares of each intensity value.

$$uniformity = \sum_{i=1}^{N_g} p(i)^2$$

2.2 Shape features

In this group of features we included descriptors of the three-dimensional size and shape of the ROI. These features are independent from the gray level intensity distribution in the ROI

and are therefore only calculated on the non-derived image and mask.

1) Mesh Volume: The volume of the ROI is calculated from the triangle mesh of the ROI. For each face i in the mesh, defined by points a_i, b_i and c_i, the (signed) volume V_f of the tetrahedron defined by that face and the origin of the image (O) is calculated. (1) The sign of the volume is determined by the sign of the normal, which must be consistently defined as either facing outward or inward of the ROI.

Then taking the sum of all V_i, the total volume of the ROI is obtained (2)

$$V_i = \frac{Oa_i \cdot (Ob_i \times Oc_i)}{6} \quad (1)$$

$$V = \sum_{i=1}^{N_f} V_i \quad (2)$$

2) Voxel Volume: The volume of the ROI V_{voxel} is approximated by multiplying the number of voxels in the ROI by the volume of a single voxel V_k. This is a less precise approximation of the volume and is not used in subsequent features. This feature does not make use of the mesh and is not used in calculation of other shape features.

$$V_{voxel} = \sum_{k=1}^{N_v} V_k$$

3) Surface Area: To calculate the surface area, first the surface area A_i of each triangle in the mesh is calculated (1). The total surface area is then obtained by taking the sum of all calculated sub-areas (2).

$$A_i = \frac{1}{2}|\text{a}_i\text{b}_i \times \text{a}_i\text{c}_i| \quad (1)$$

$$A = \sum_{i=1}^{N_f} A_i \quad (2)$$

where:

a_ib_i and a_ic_i are edges of the ith triangle in the mesh, formed by vertices a_i, b_i, c_i.

4) Surface Area to Volume ratio: Here, a lower value indicates a more compact (sphere-like) shape. This feature is not dimensionless, and is therefore (partly) dependent on the volume of the ROI.

$$surface\ to\ volume\ ratio = \frac{A}{V}$$

5) Sphericity: Sphericity is a measure of the roundness of the shape of the tumor region relative to a sphere. It is a dimensionless measure, independent of scale and orientation. The value range is $0 \le \text{sphericity} \le 1$, where a value of 1 indicates a perfect sphere (a sphere has the smallest possible surface area for a given volume, compared to other solids).

$$sphericity = \frac{\sqrt[3]{36\pi V^2}}{A}$$

6) Maximum 3D diameter: Maximum 3D diameter is defined as the largest pairwise Euclidean distance between tumor surface mesh vertices.

7) Maximum 2D diameter (Slice): Maximum 2D diameter (Slice) is defined as the largest pairwise Euclidean distance between tumor surface mesh vertices in the row-column (generally the axial) plane.

8) Maximum 2D diameter (Column): Maximum 2D diameter (Column) is defined as the largest pairwise Euclidean distance between tumor surface mesh vertices in the row-slice (usually the coronal) plane.

9) Maximum 2D diameter (Row): Maximum 2D diameter (Row) is defined as the largest pairwise Euclidean distance between tumor surface mesh vertices in the column-slice (usually the sagittal) plane.

10) Major Axis Length: This feature yield the largest axis length of the ROI-enclosing ellipsoid and is calculated using the largest principal component λ_{major} . The principal component analysis is performed using the physical coordinates of the voxel centers defining the ROI. It therefore takes spacing into account, but does not make use of the shape mesh.

$$major\ axis = 4\sqrt{\lambda_{major}}$$

11) Minor Axis Length: This feature yield the second-largest axis length of the ROI-enclosing ellipsoid and is calculated using the largest principal component λ_{minor} . The principal component analysis is performed using the physical coordinates of the voxel centers defining the ROI. It therefore takes spacing into account, but does not make use of the shape mesh.

$$minor\ axis = 4\sqrt{\lambda_{minor}}$$

12) Least Axis Length: This feature yield the smallest axis length of the ROI-enclosing ellipsoid and is calculated using the largest principal component λ_{least} . In case of a 2D segmentation, this value will be 0. The principal component analysis is performed using the physical coordinates of the voxel centers defining the ROI. It therefore takes spacing into account, but does not make use of the shape mesh.

$$least\ axis = 4\sqrt{\lambda_{least}}$$

13) Elongation: Elongation shows the relationship between the two largest principal components in the ROI shape. Here, λ_{major} and λ_{minor} are the lengths of the largest and second largest principal component axes. The values range between 1 (where the cross section through the first and second largest principal moments is circle-like (non-elongated)) and 0 (where the object is a maximally elongated: i.e. a 1 dimensional line). The principal component analysis is performed using the physical coordinates of the voxel centers defining the ROI. It therefore takes spacing into account, but does not make use of the shape mesh.

$$elongation = \sqrt{\frac{\lambda_{minor}}{\lambda_{major}}}$$

14) Flatness: Flatness shows the relationship between the largest and smallest principal components in the ROI shape. For computational reasons, this feature is defined as the inverse of true flatness. Here, λ_{major} and λ_{least} are the lengths of the largest and smallest principal component axes. The values range between 1 (non-flat, sphere-like) and 0 (a flat object, or single-slice segmentation). The principal component analysis is performed using the physical coordinates of the voxel centers defining the ROI. It therefore takes spacing into account, but does not make use of the shape mesh.

$$flatness = \sqrt{\frac{\lambda_{least}}{\lambda_{major}}}$$

2.3 Gray Level Co-occurrence Matrix (GLCM) features

A Gray Level Co-occurrence Matrix (GLCM) of size $N_g \times N_g$ describes the second-order joint probability function of an image region constrained by the mask and is defined as $P(i,j|\delta,\theta)$. The $(i,j)^{th}$ element of this matrix represents the number of times the combination of levels i and j occur in two pixels in the image, that are separated by a distance of δ pixels along angle θ. The distance δ from the center voxel is defined as the distance according to the infinity norm. For $\delta = 1$, this results in 2 neighbors for each of 13 angles in 3D (26-connectivity) and for a 98-connectivity (49 unique angles).

1) Autocorrelation: Autocorrelation is a measure of the magnitude of the fineness and coarseness of texture.

$$autocorrelation = \sum_{i=1}^{N_g}\sum_{j=1}^{N_g} p(i,j)ij$$

2) Joint Average: Returns the mean gray level intensity of the i distribution.

$$joint\ average = \mu_x = \sum_{i=1}^{N_g}\sum_{j=1}^{N_g} p(i,j)i$$

3) Cluster Prominence: Cluster Prominence is a measure of the skewness and asymmetry of the GLCM.

$$cluster\ prominence = \sum_{i=1}^{N_g}\sum_{j=1}^{N_g} \left(i + j - \mu_x - \mu_y\right)^4 p(i,j)$$

4) Cluster Shade: Cluster Shade is a measure of the skewness and uniformity of the GLCM.

$$cluster\ shade = \sum_{i=1}^{N_g}\sum_{j=1}^{N_g} \left(i + j - \mu_x - \mu_y\right)^3 p(i,j)$$

5) Cluster Tendency: Cluster Tendency is a measure of groupings of voxels with similar gray-level values.

$$cluster\ tendency = \sum_{i=1}^{N_g}\sum_{j=1}^{N_g} \left(i + j - \mu_x - \mu_y\right)^2 p(i,j)$$

6) Contrast: Contrast is a measure of the local intensity variation, favoring values away from the diagonal $i = j$. A larger value correlates with a greater disparity in intensity values among neighboring voxels.

$$contrast = \sum_{i=1}^{N_g}\sum_{j=1}^{N_g} (i - j)^2 p(i,j)$$

7) Correlation: Correlation is a value between 0 (uncorrelated) and 1 (perfectly correlated) showing the linear dependency of gray level values to their respective voxels in the GLCM.

$$correlation = \frac{\sum_{i=1}^{N_g}\sum_{j=1}^{N_g} p(i,j)ij - \mu_x\mu_y}{\sigma_x(i)\sigma_y(j)}$$

8) Difference Average: Difference Average measures the relationship between occurrences of pairs with similar intensity values and occurrences of pairs with differing intensity values.

$$difference\ average = \sum_{k=0}^{N_g-1} k p_{x-y}(k)$$

9) Difference Entropy: Difference Entropy is a measure of the randomness/variability in neighborhood intensity value differences.

$$difference\ entropy = \sum_{k=0}^{N_g-1} p_{x-y}(k) \log_2 \big(p_{x-y}(k) + \epsilon\big)$$

10) Difference Variance: Difference Variance is a measure of heterogeneity that places higher weights on differing intensity level pairs that deviate more from the mean.

$$difference\ variance = \sum_{k=0}^{N_g-1} (k - DA)^2 p_{x-y}(k)$$

11) Joint Energy: Energy is a measure of homogeneous patterns in the image.

$$joint\ energy = \sum_{i=1}^{N_g} \sum_{j=1}^{N_g} \big(p(i,j)\big)^2$$

12) Joint Entropy: Joint entropy is a measure of the randomness/variability in neighborhood intensity values.

$$joint\ entropy = -\sum_{i=1}^{N_g} \sum_{j=1}^{N_g} p(i,j) \log_2 \big(p(i,j) + \epsilon\big)$$

13) Informational Measure of Correlation (IMC) 1:

$$IMC\ 1 = \frac{HXY - HXY1}{\max\{HX, HY\}}$$

14) Informational Measure of Correlation (IMC) 2:

$$IMC\ 2 = \sqrt{1 - e^{-2(HXY2 - HXY)}}$$

15) Inverse Difference Moment (IDM): IDM (a.k.a Homogeneity 2) is a measure of the local homogeneity of an image. IDM weights are the inverse of the Contrast weights (decreasing exponentially from the diagonal i=j in the GLCM).

$$IDM = \sum_{k=0}^{N_g-1} \frac{p_{x-y}(k)}{1+k^2}$$

16) Maximal Correlation Coefficient (MCC): The Maximal Correlation Coefficient is a measure of complexity of the texture and $0 \leq MCC \leq 1$.

$$MCC = \sqrt{\text{second largest eigenvalue of Q}}$$

$$Q(i,j) = \sum_{k=0}^{N_g} \frac{p(i,k)p(j,k)}{p_x(i)p_y(k)}$$

17) Inverse Difference Moment Normalized (IDMN): IDMN (inverse difference moment normalized) is a measure of the local homogeneity of an image. IDMN weights are the inverse of the Contrast weights (decreasing exponentially from the diagonal $i = j$ in the GLCM). Unlike Homogeneity2, IDMN normalizes the square of the difference between neighboring intensity values by dividing over the square of the total number of discrete intensity values.

$$IDMN = \sum_{k=0}^{N_g-1} \frac{p_{x-y}(k)}{1+\left(\frac{k^2}{N_g^2}\right)}$$

18) Inverse Difference (ID): ID (a.k.a. Homogeneity 1) is another measure of the local homogeneity of an image. With more uniform gray levels, the denominator will remain low, resulting in a higher overall value.

$$ID = \sum_{k=0}^{N_g-1} \frac{p_{x-y}(k)}{1+k}$$

19) Inverse Difference Normalized (IDN): IDN (inverse difference normalized) is another measure of the local homogeneity of an image.

$$IDN = \sum_{k=0}^{N_g-1} \frac{p_{x-y}(k)}{1+\left(\frac{k}{N_g}\right)}$$

20) Inverse Variance:

$$\mathit{inverse\ variance} = \sum_{k=1}^{N_g-1} \frac{p_{x-y}(k)}{k^2}$$

21) Maximum Probability: Maximum Probability is occurrences of the most predominant

pair of neighboring intensity values.

$$maximum\ probability = \max\big(p(i,j)\big)$$

22) Sum Average: Sum Average measures the relationship between occurrences of pairs with lower intensity values and occurrences of pairs with higher intensity values.

$$sum\ average = \sum_{k=2}^{2N_g} p_{x+y}(k)k$$

23) Sum Entropy: Sum Entropy is a sum of neighborhood intensity value differences.

$$sum\ entropy = \sum_{k=2}^{2N_g} p_{x+y}(k)\log_2\big(p_{x+y}(k)+\epsilon\big)$$

24) Sum of Squares: Sum of Squares or Variance is a measure in the distribution of neigboring intensity level pairs about the mean intensity level in the GLCM.

$$sum\ squares = \sum_{i=1}^{N_g}\sum_{j=1}^{N_g}(i-\mu_x)^2 p(i,j)$$

2.4 Gray Level Run Length Matrix (GLRLM) features

A Gray Level Run Length Matrix (GLRLM) quantifies gray level runs, which are defined as the length in number of pixels, of consecutive pixels that have the same gray level value. In a gray level run length matrix $P(i,j\,|\,\theta)$, the $(i,j)^{th}$ element describes the number of runs with gray level i and length occur in the image (ROI) along angle θ.

1) Short Run Emphasis (SRE): SRE is a measure of the distribution of short run lengths, with a greater value indicative of shorter run lengths and more fine textural textures.

$$SRE = \frac{\sum_{i=1}^{N_g}\sum_{j=1}^{N_r}\frac{\mathbf{P}(i,j|\theta)}{j^2}}{N_r(\theta)}$$

2) Long Run Emphasis (LRE): LRE is a measure of the distribution of long run lengths, with a greater value indicative of longer run lengths and more coarse structural textures.

$$LRE = \frac{\sum_{i=1}^{N_g}\sum_{j=1}^{N_r}\mathbf{P}(i,j|\theta)j^2}{N_r(\theta)}$$

3) Gray Level Non-Uniformity (GLN): GLN measures the similarity of gray-level intensity

values in the image, where a lower GLN value correlates with a greater similarity in intensity values.

$$GLN = \frac{\sum_{i=1}^{N_g} \left(\sum_{j=1}^{N_r} \mathbf{P}(i,j|\theta) \right)^2}{N_r(\theta)}$$

4) Gray Level Non-Uniformity Normalized (GLNN): GLNN measures the similarity of gray-level intensity values in the image, where a lower GLNN value correlates with a greater similarity in intensity values.

$$GLNN = \frac{\sum_{i=1}^{N_g} \left(\sum_{j=1}^{N_r} \mathbf{P}(i,j|\theta) \right)^2}{N_r(\theta)^2}$$

5) Run Length Non-Uniformity (RLN): RLN measures the similarity of run lengths throughout the image, with a lower value indicating more homogeneity among run lengths in the image.

$$RLN = \frac{\sum_{j=1}^{N_r} \left(\sum_{i=1}^{N_g} \mathbf{P}(i,j|\theta) \right)^2}{N_r(\theta)}$$

6) Run Length Non-Uniformity Normalized (RLNN): RLNN measures the similarity of run lengths throughout the image, with a lower value indicating more homogeneity among run lengths in the image.

$$RLNN = \frac{\sum_{j=1}^{N_r} \left(\sum_{i=1}^{N_g} \mathbf{P}(i,j|\theta) \right)^2}{N_r(\theta)^2}$$

7) Run Percentage (RP): RP measures the coarseness of the texture by taking the ratio of number of runs and number of voxels in the ROI.

$$RP = \frac{N_r(\theta)}{N_p}$$

8) Gray Level Variance (GLV): GLV measures the variance in gray level intensity for the runs.

$$GLV = \sum_{i=1}^{N_g} \sum_{j=1}^{N_r} p(i,j|\theta)(i-\mu)^2$$

9) Run Variance (RV): RV is a measure of the variance in runs for the run lengths.

$$RV = \sum_{i=1}^{N_g} \sum_{j=1}^{N_r} p(i,j|\theta)(j-\mu)^2$$

10) Run Entropy (RE): Here, ε is an arbitrarily small positive number $\approx (2.2\times10^{-16})$. RE measures the uncertainty/randomness in the distribution of run lengths and gray levels.

$$RE = -\sum_{i=1}^{N_g} \sum_{j=1}^{N_r} p(i,j|\theta) \log_2(p(i,j|\theta)+\epsilon)$$

11) Low Gray Level Run Emphasis (LGLRE): LGLRE measures the distribution of low gray-level values, with a higher value indicating a greater concentration of low gray-level values in the image.

$$LGLRE = \frac{\sum_{i=1}^{N_g} \sum_{j=1}^{N_r} \frac{\mathbf{P}(i,j|\theta)}{i^2}}{N_r(\theta)}$$

12) High Gray Level Run Emphasis (HGLRE): HGLRE measures the distribution of the higher gray-level values, with a higher value indicating a greater concentration of high gray-level values in the image.

$$HGLRE = \frac{\sum_{i=1}^{N_g} \sum_{j=1}^{N_r} \mathbf{P}(i,j|\theta) i^2}{N_r(\theta)}$$

13) Short Run Low Gray Level Emphasis (SRLGLE): SRLGLE measures the joint distribution of shorter run lengths with lower gray-level values.

$$SRLGLE = \frac{\sum_{i=1}^{N_g} \sum_{j=1}^{N_r} \frac{\mathbf{P}(i,j|\theta)}{i^2 j^2}}{N_r(\theta)}$$

14) Short Run High Gray Level Emphasis (SRHGLE): SRHGLE measures the joint distribution of shorter run lengths with higher gray-level values.

$$SRHGLE = \frac{\sum_{i=1}^{N_g} \sum_{j=1}^{N_r} \frac{\mathbf{P}(i,j|\theta) i^2}{j^2}}{N_r(\theta)}$$

15) Long Run Low Gray Level Emphasis (LRLGLE): LRLGLRE measures the joint distribution of long run lengths with lower gray-level values.

$$LRLGLRE = \frac{\sum_{i=1}^{N_g}\sum_{j=1}^{N_r}\frac{\mathbf{P}(i,j|\theta)j^2}{i^2}}{N_r(\theta)}$$

16) Long Run High Gray Level Emphasis (LRHGLE): LRHGLRE measures the joint distribution of long run lengths with higher gray-level values.

$$LRHGLRE = \frac{\sum_{i=1}^{N_g}\sum_{j=1}^{N_r}\mathbf{P}(i,j|\theta)i^2j^2}{N_r(\theta)}$$

2.5 Neighbouring Gray Tone Difference Matrix (NGTDM) features

A Neighbouring Gray Tone Difference Matrix quantifies the difference between a gray value and the average gray value of its neighbours within distance δ . The sum of absolute differences for gray level is stored in the matrix.

1) Coarseness: Coarseness is a measure of average difference between the center voxel and its neighbourhood and is an indication of the spatial rate of change.

$$Coarseness = \frac{1}{\sum_{i=1}^{N_g}p_is_i}$$

2) Contrast: Contrast is a measure of the spatial intensity change, but is also dependent on the overall gray level dynamic range.

$$Contrast = \left(\frac{1}{N_{g,p}(N_{g,p}-1)}\sum_{i=1}^{N_g}\sum_{j=1}^{N_g}p_ip_j(i-j)^2\right)\left(\frac{1}{N_{v,p}}\sum_{i=1}^{N_g}s_i\right)$$

where $p_i \neq 0, p_j \neq 0$.

3) Busyness: A measure of the change from a pixel to its neighbour.

$$Busyness = \frac{\sum_{i=1}^{N_g}p_is_i}{\sum_{i=1}^{N_g}\sum_{j=1}^{N_g}|ip_i - jp_j|}$$

where $p_i \neq 0, p_j \neq 0$.

4) Complexity: An image is considered complex when there are many primitive components in the image, i.e. the image is non-uniform and there are many rapid changes in gray level intensity.

$$Complexity = \frac{1}{N_{v,p}} \sum_{i=1}^{N_g} \sum_{j=1}^{N_g} |i-j| \frac{p_i s_i + p_j s_j}{p_i + p_j}$$

where $p_i \neq 0, p_j \neq 0$.

5) Strength: Strength is a measure of the primitives in an image. Its value is high when the primitives are easily defined and visible, i.e. an image with slow change in intensity but more large coarse differences in gray level intensities.

$$Strength = \frac{\sum_{i=1}^{N_g} \sum_{j=1}^{N_g} (p_i + p_j)(i-j)^2}{\sum_{i=1}^{N_g} s_i}$$

where $p_i \neq 0, p_j \neq 0$.

2.6 Gray Level Dependence Matrix (GLDM) features

A Gray Level Dependence Matrix (GLDM) quantifies gray level dependencies in an image. A gray level dependency is defined as a the number of connected voxels within distance that are dependent on the center voxel. A neighbouring voxel with gray level j is considered dependent on center voxel with gray level i if $|i-j| \leq \alpha$. In a gray level dependence matrix $P(i,j)$ the $(i,j)^{th}$ element describes the number of times a voxel with gray level i with j dependent voxels in its neighbourhood appears in image.

1) Small Dependence Emphasis (SDE): A measure of the distribution of small dependencies, with a greater value indicative of smaller dependence and less homogeneous textures.

$$SDE = \frac{\sum_{i=1}^{N_g} \sum_{j=1}^{N_d} \frac{\mathbf{P}(i,j)}{i^2}}{N_z}$$

2) Large Dependence Emphasis (LDE): A measure of the distribution of large dependencies, with a greater value indicative of larger dependence and more homogeneous textures.

$$LDE = \frac{\sum_{i=1}^{N_g} \sum_{j=1}^{N_d} \mathbf{P}(i,j) j^2}{N_z}$$

3) Gray Level Non-Uniformity (GLN): Measures the similarity of gray-level intensity values in the image, where a lower GLN value correlates with a greater similarity in intensity values.

$$GLN = \frac{\sum_{i=1}^{N_g} \left(\sum_{j=1}^{N_d} \mathbf{P}(i,j) \right)^2}{N_z}$$

4) Dependence Non-Uniformity (DN): Measures the similarity of dependence throughout the image, with a lower value indicating more homogeneity among dependencies in the image.

$$DN = \frac{\sum_{j=1}^{N_d} \left(\sum_{i=1}^{N_g} \mathbf{P}(i,j) \right)^2}{N_z}$$

5) Dependence Non-Uniformity Normalized (DNN): Measures the similarity of dependence throughout the image, with a lower value indicating more homogeneity among dependencies in the image.

$$DNN = \frac{\sum_{j=1}^{N_d} \left(\sum_{i=1}^{N_g} \mathbf{P}(i,j) \right)^2}{N_z^2}$$

6) Gray Level Variance (GLV): Measures the variance in gray level in the image.

$$GLV = \sum_{i=1}^{N_g} \sum_{j=1}^{N_d} p(i,j)(i-\mu)^2, \text{ where} \mu = \sum_{i=1}^{N_g} \sum_{j=1}^{N_d} ip(i,j)$$

7) Dependence Variance (DV): Measures the variance in dependence size in the image.

$$DV = \sum_{i=1}^{N_g} \sum_{j=1}^{N_d} p(i,j)(j-\mu)^2, \text{ where} \mu = \sum_{i=1}^{N_g} \sum_{j=1}^{N_d} jp(i,j)$$

8) Dependence Entropy (DE):

$$DependenceEntropy = -\sum_{i=1}^{N_g} \sum_{j=1}^{N_d} p(i,j) \log_2 (p(i,j) + \epsilon)$$

9) Low Gray Level Emphasis (LGLE): Measures the distribution of low gray-level values, with a higher value indicating a greater concentration of low gray-level values in the image.

$$LGLE = \frac{\sum_{i=1}^{N_g} \sum_{j=1}^{N_d} \frac{\mathbf{P}(i,j)}{i^2}}{N_z}$$

10) High Gray Level Emphasis (HGLE): Measures the distribution of the higher gray-level values, with a higher value indicating a greater concentration of high gray-level values in the image.

$$HGLE = \frac{\sum_{i=1}^{N_g} \sum_{j=1}^{N_d} \mathbf{P}(i,j) i^2}{N_z}$$

11) Small Dependence Low Gray Level Emphasis (SDLGLE): Measures the joint distribution of small dependence with lower gray-level values.

$$SDLGLE = \frac{\sum_{i=1}^{N_g} \sum_{j=1}^{N_d} \frac{\mathbf{P}(i,j)}{i^2 j^2}}{N_z}$$

12) Small Dependence High Gray Level Emphasis (SDHGLE): Measures the joint distribution of small dependence with lower gray-level values.

$$SDLGLE = \frac{\sum_{i=1}^{N_g} \sum_{j=1}^{N_d} \frac{\mathbf{P}(i,j)}{i^2 j^2}}{N_z}$$

13) Large Dependence Low Gray Level Emphasis (LDLGLE): Measures the joint distribution of large dependence with lower gray-level values.

$$LDLGLE = \frac{\sum_{i=1}^{N_g} \sum_{j=1}^{N_d} \frac{\mathbf{P}(i,j) j^2}{i^2}}{N_z}$$

14) Large Dependence High Gray Level Emphasis (LDHGLE): Measures the joint distribution of large dependence with higher gray-level values.

$$LDHGLE = \frac{\sum_{i=1}^{N_g} \sum_{j=1}^{N_d} \mathbf{P}(i,j) i^2 j^2}{N_z}$$

2.7 Gray Level Size Zone Matrix (GLSZM) features

A Gray Level Size Zone (GLSZM) quantifies gray level zones in an image. A gray level zone is defined as a the number of connected voxels that share the same gray level intensity. A voxel is considered connected if the distance is 1 according to the infinity norm (26-connected region in a 3D, 8-connected region in 2D). In a gray level size zone matrix $P(i, j)$ the $(i, j)^{th}$ element equals the number of zones with gray level i and size j appear in image. Contrary to GLCM and GLRLM, the GLSZM is rotation independent, with only one matrix calculated for all directions in the ROI.

1) Small Area Emphasis (SAE): SAE is a measure of the distribution of small size zones, with a greater value indicative of more smaller size zones and more fine textures.

$$SAE = \frac{\sum_{i=1}^{N_g} \sum_{j=1}^{N_s} \frac{\mathbf{P}(i,j)}{j^2}}{N_z}$$

2) Large Area Emphasis (LAE): LAE is a measure of the distribution of large area size zones, with a greater value indicative of more larger size zones and more coarse textures.

$$LAE = \frac{\sum_{i=1}^{N_g} \sum_{j=1}^{N_s} \mathbf{P}(i,j) j^2}{N_z}$$

3) Gray Level Non-Uniformity (GLN): GLN measures the variability of gray-level intensity values in the image, with a lower value indicating more homogeneity in intensity values.

$$GLN = \frac{\sum_{i=1}^{N_g} \left(\sum_{j=1}^{N_s} \mathbf{P}(i,j) \right)^2}{N_z}$$

4) Gray Level Non-Uniformity Normalized (GLNN): GLNN measures the variability of gray-level intensity values in the image, with a lower value indicating a greater similarity in intensity values.

$$GLNN = \frac{\sum_{i=1}^{N_g} \left(\sum_{j=1}^{N_s} \mathbf{P}(i,j) \right)^2}{N_z^2}$$

5) Size-Zone Non-Uniformity (SZN): SZN measures the variability of size zone volumes in the image, with a lower value indicating more homogeneity in size zone volumes.

$$SZN = \frac{\sum_{j=1}^{N_s} \left(\sum_{i=1}^{N_g} \mathbf{P}(i,j) \right)^2}{N_z}$$

6) Size-Zone Non-Uniformity Normalized (SZNN): SZNN measures the variability of size zone volumes throughout the image, with a lower value indicating more homogeneity among zone size volumes in the image.

$$SZNN = \frac{\sum_{j=1}^{N_s} \left(\sum_{i=1}^{N_g} \mathbf{P}(i,j) \right)^2}{N_z^2}$$

7) Zone Percentage (ZP): ZP measures the coarseness of the texture by taking the ratio of

number of zones and number of voxels in the ROI.

$$ZP = \frac{N_z}{N_p}$$

8) Gray Level Variance (GLV): GLV measures the variance in gray level intensities for the zones.

$$GLV = \sum_{i=1}^{N_g} \sum_{j=1}^{N_s} p(i,j)(i-\mu)^2$$

9) Zone Variance (ZV): ZV measures the variance in zone size volumes for the zones.

$$ZV = \sum_{i=1}^{N_g} \sum_{j=1}^{N_s} p(i,j)(j-\mu)^2$$

10) Zone Entropy (ZE): Here, ε is an arbitrarily small positive number $\approx (2.2\times10^{-16})$. ZE measures the uncertainty/randomness in the distribution of zone sizes and gray levels.

$$ZE = -\sum_{i=1}^{N_g} \sum_{j=1}^{N_s} p(i,j) \log_2(p(i,j)+\epsilon)$$

11) Low Gray Level Zone Emphasis (LGLZE): LGLZE measures the distribution of lower gray-level size zones, with a higher value indicating a greater proportion of lower gray-level values and size zones in the image.

$$LGLZE = \frac{\sum_{i=1}^{N_g} \sum_{j=1}^{N_s} \frac{\mathbf{P}(i,j)}{i^2}}{N_z}$$

12) High Gray Level Zone Emphasis (HGLZE): HGLZE measures the distribution of the higher gray-level values, with a higher value indicating a greater proportion of higher gray-level values and size zones in the image.

$$HGLZE = \frac{\sum_{i=1}^{N_g} \sum_{j=1}^{N_s} \mathbf{P}(i,j) i^2}{N_z}$$

13) Small Area Low Gray Level Emphasis (SALGLE): SALGLE measures the proportion in the image of the joint distribution of smaller size zones with lower gray-level values.

$$SALGLE = \frac{\sum_{i=1}^{N_g} \sum_{j=1}^{N_s} \frac{\mathbf{P}(i,j)}{i^2 j^2}}{N_z}$$

14) Small Area High Gray Level Emphasis (SAHGLE): SAHGLE measures the proportion in the image of the joint distribution of smaller size zones with higher gray-level values.

$$SAHGLE = \frac{\sum_{i=1}^{N_g} \sum_{j=1}^{N_s} \frac{\mathbf{P}(i,j) i^2}{j^2}}{N_z}$$

15) Large Area Low Gray Level Emphasis (LALGLE): LALGLE measures the proportion in the image of the joint distribution of larger size zones with lower gray-level values.

$$LALGLE = \frac{\sum_{i=1}^{N_g} \sum_{j=1}^{N_s} \frac{\mathbf{P}(i,j) j^2}{i^2}}{N_z}$$

16) Large Area High Gray Level Emphasis (LAHGLE): LAHGLE measures the proportion in the image of the joint distribution of larger size zones with higher gray-level values.

$$LAHGLE = \frac{\sum_{i=1}^{N_g} \sum_{j=1}^{N_s} \mathbf{P}(i,j) i^2 j^2}{N_z}$$

2.8 Wavelet features

Wavelet filtering, yields 8 decompositions per level (all possible combinations of applying either a High or a Low pass filter in each of the three dimensions).

2.9 Laplacian of Gaussian (LOG) features

Laplacian of Gaussian filter, edge enhancement filter. Emphasizes areas of gray level change, where sigma defines how coarse the emphasised texture should be. A low sigma emphasis on fine textures (change over a short distance), where a high sigma value emphasises coarse textures (gray level change over a large distance).

S3. Detailed diagram depicting the fusion network architecture

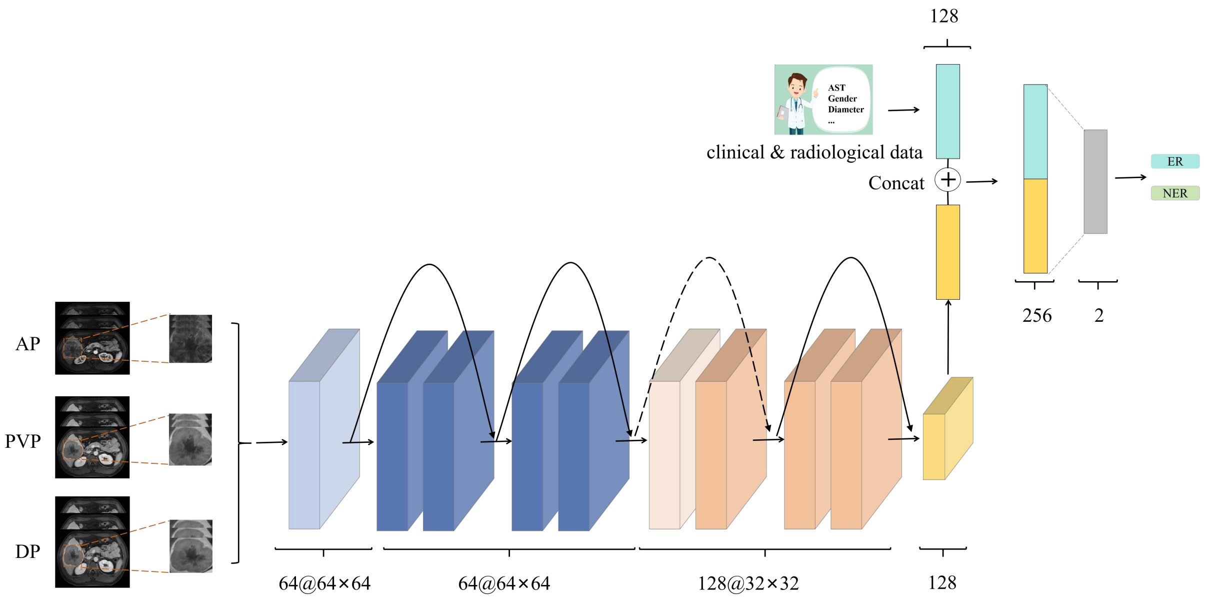

Figure S3. The network architecture of the deep learning combined with radiomics and clinical-radiological (DLRCR) model. The contrast enhanced MR images from three phases (arterial phase (AP), portal venous phase (PVP), and delayed phase (DP)) were concatenated and fed into the convolutional layers for feature extraction. Selected radiomics features and clinical-radiological risk factors were encoded and input into a multilayer perceptron (MLP) with one fully connected layer. This MLP output was then concatenated with the image features to predict early recurrence.

S4. The selected radiomics features and the corresponding coefficients

Phase	Feature	Coefficient
AP	log-sigma-1-0-mm-3D_glcm_Imc1	-0.006559768
	log-sigma-1-0-mm-3D_glrlm_ShortRunLowGrayLevelEmphasis	-0.01516737
	log-sigma-3-0-mm-3D_glcm_InverseVariance	-0.003227009
	log-sigma-3-0-mm-3D_glrlm_RunVariance	0.026149502
	original_firstorder_Skewness	0.030584796
	wavelet-HHH_glszm_SmallAreaHighGrayLevelEmphasis	0.012150321
	wavelet-HHL_glrlm_LongRunHighGrayLevelEmphasis	0.018401961
	wavelet-HHL_glszm_SmallAreaHighGrayLevelEmphasis	0.008471705
	wavelet-HLH_firstorder_InterquartileRange	-0.012820349
	wavelet-HLL_glszm_LargeAreaEmphasis	0.008966625
	wavelet-LHH_glcm_MaximumProbability	-0.090247348
	wavelet-LLH_firstorder_Maximum	0.07642916
	wavelet-LLH_glszm_LargeAreaEmphasis	0.001258987
	wavelet-LLH_glszm_LargeAreaLowGrayLevelEmphasis	0.000165338
PVP	log-sigma-1-0-mm-3D_glcm_Correlation	0.000187089
	log-sigma-3-0-mm-3D_glcm_InverseVariance	-0.021286646
	wavelet-HHH_glszm_GrayLevelNonUniformity	0.029235194
	wavelet-HLH_glcm_JointEnergy	-0.041546663
	wavelet-LHH_glcm_JointEnergy	-0.006395344
	wavelet-LHH_glszm_LargeAreaLowGrayLevelEmphasis	0.016857535
	wavelet-LHL_glcm_JointAverage	0.009061645
	wavelet-LLH_firstorder_RootMeanSquared	0.021763875
	wavelet-LLH_glcm_Imc1	-0.029267346
	wavelet-LLH_gldm_LargeDependenceLowGrayLevelEmphasis	-0.030005372
	wavelet-LLH_glrlm_RunEntropy	0.012415092
	wavelet-LLH_ngtdm_Coarseness	-0.009866386
	wavelet-LLL_firstorder_Minimum	-0.015735357
DP	log-sigma-1-0-mm-3D_glcm_MCC	0.034418748
	log-sigma-1-0-mm-3D_glrlm_LongRunHighGrayLevelEmphasis	0.036848973
	wavelet-HHH_glszm_GrayLevelNonUniformity	0.012037863
	wavelet-HLL_glszm_ZoneVariance	0.014692736
	wavelet-LHH_glcm_JointEnergy	-0.048843637
	wavelet-LHH_ngtdm_Busyness	0.003065251
	wavelet-LLH_glcm_Correlation	-0.028681923

AP, arterial phase; PVP, portal venous phase; DP, delayed phase.

The top-ranked radiomics features were composed of: 1 histogram-based feature, 8 Laplacian of Gaussian features (5 glcm features and 3 glrlm features), and 25 wavelet features (4 histogram-based features, 7 glcm features, 2 glrlm features, 2 ngtdm features, 1 gldm feature, and 9 glszm features). Numerous studies have showed that these features describe the distribution of voxel intensities within an image and the spatial relationship between local nearby pixels, and they have served as recognized parameters to reflect tumor heterogeneity. Previous studies[1-3] have demonstrated that HCC recurrence is closely related to tumor heterogeneity identified by imaging radiomics features.

S5. ROC curves of five machine learning classifiers in the training cohort (A) and the validation cohort (B).

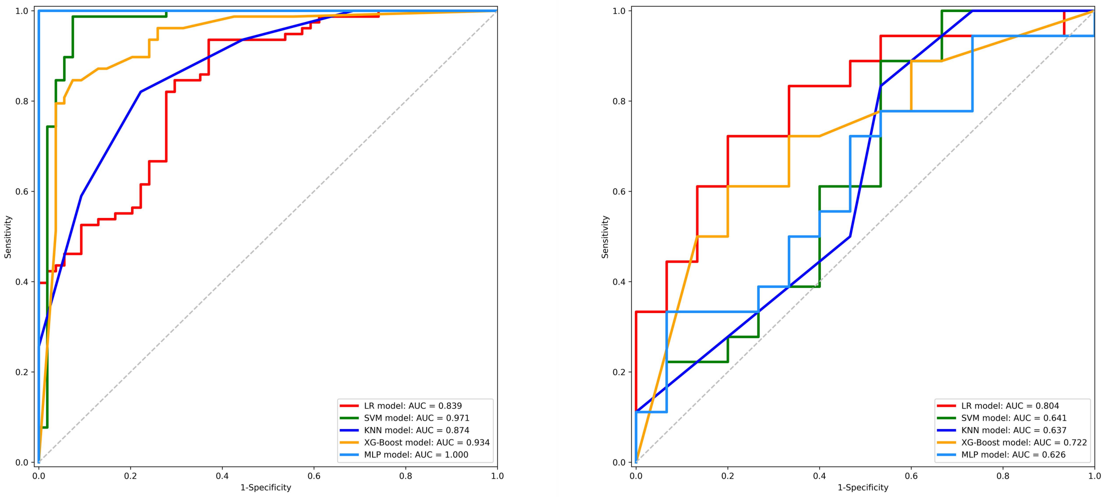

S6. Predictive performances and ROC curves of DL models

Table S6. Predictive performances of DL models in the training and validation cohorts

Model		AUC (95%CI)	Accuracy	Sensitivity	Specificity
ResNet-10	Training cohort	0.870 (0.806 - 0.934)	0.803	0.861	0.733
	Validation cohort	0.826 (0.682 - 0.970)	0.788	0.824	0.750
ResNet-18	Training cohort	0.700 (0.608 - 0.792)	0.652	0.645	0.682
	Validation cohort	0.704 (0.517 - 0.890)	0.727	0.696	0.800
ResNet-34	Training cohort	0.611 (0.511 - 0.712)	0.644	0.642	0.652
	Validation cohort	0.619 (0.414 - 0.823)	0.606	0.593	0.667

DL, deep learning; AUC, area under the curve; CI, confidence interval.

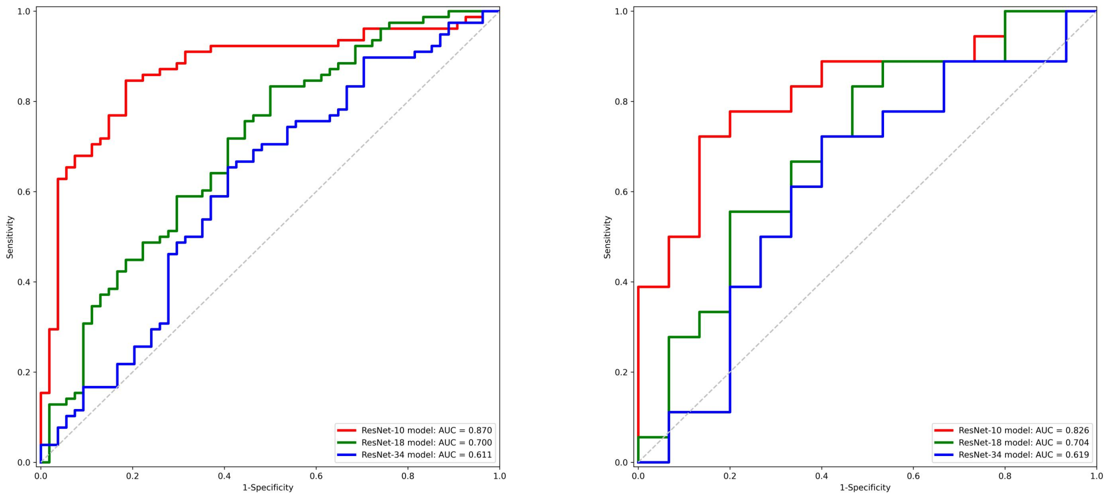

Figure S6. ROC curves of DL models in the training (A) and validation cohorts (B).

S7. Delong's test of different predictive models in the training and validation cohorts

Validation / Training	CR model	Radiomics model	DL model	RCR model	DLRCR model
CR model	—	0.515	0.448	0.440	0.232
Radiomics model	6.892e-05	—	0.857	0.527	0.423
DL model	0.0002075	0.509	—	0.887	0.857
RCR model	4.963e-05	0.291	0.538	—	0.527
DLRCR model	2.357e-06	0.080	0.275	0.090	—

CR, clinical-radiological; DL, deep learning; RCR, radiomics combined with clinical-radiological; DLRCR, deep learning combined with RCR.

S8. Predictive performances and ROC curves of clinical prediction methods

Table S8. Predictive performances of clinical prediction methods in the both cohorts

Model		AUC (95%CI)	Accuracy	Sensitivity	Specificity
BCLC system	Training cohort	0.565 (0.507 - 0.623)	0.621	0.974	0.111
	Validation cohort	0.615 (0.513 - 0.717)	0.606	1.000	0.133
CNLC system	Training cohort	0.610 (0.529 - 0.692)	0.583	0.474	0.741
	Validation cohort	0.774 (0.635 - 0.914)	0.758	0.667	0.867
preoperative ERASL model	Training cohort	0.550 (0.485 - 0.616)	0.492	0.231	0.870
	Validation cohort	0.572 (0.433 - 0.711)	0.545	0.278	0.867
NLR	Training cohort	0.561 (0.476 - 0.646)	0.545	0.474	0.648
	Validation cohort	0.483 (0.307 - 0.660)	0.485	0.500	0.467
PLR	Training cohort	0.544 (0.462 - 0.626)	0.515	0.385	0.704
	Validation cohort	0.467 (0.296 - 0.637)	0.455	0.333	0.600
LMR	Training cohort	0.507 (0.422 - 0.592)	0.485	0.385	0.630
	Validation cohort	0.489 (0.313 - 0.665)	0.485	0.444	0.533

BCLC, Barcelona Clinic Liver Cancer; CNLC, Chinese National Liver Cancer; ERASL, Early Recurrence After Surgery for Liver Tumor; NLR, neutrophil-to-lymphocyte ratio; PLR, platelet-to-lymphocyte ratio; LMR, lymphocyte-to-monocyte ratio; AUC, area under the curve; CI, confidence interval.

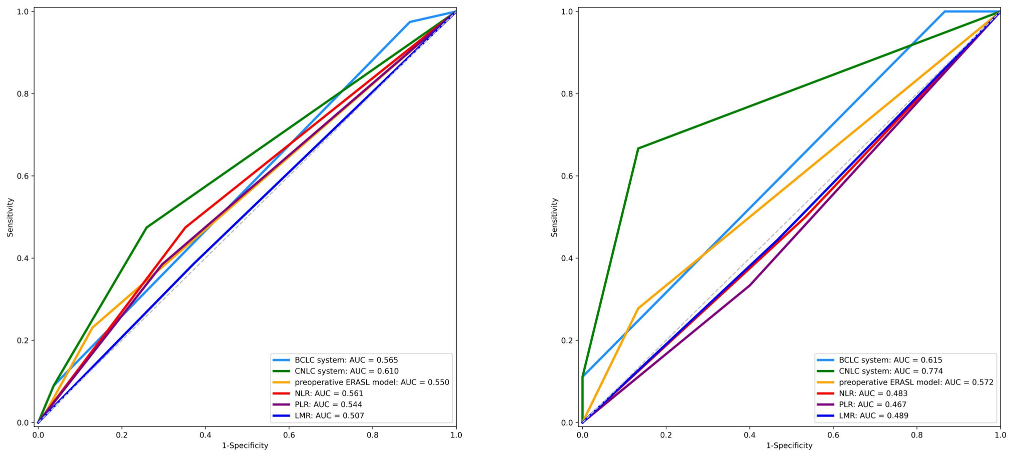

Figure S8. ROC curves of clinical prediction methods in the training (A) and validation cohorts (B).

S9. References

[1]Wei H, Zheng T, Zhang X, Wu Y, Chen Y, Zheng C, et al. MRI radiomics based on deep learning automated segmentation to predict early recurrence of hepatocellular carcinoma. Insights Imaging. (2024) 15:120. doi: 10.1186/s13244-024-01679-8.
[2]Li W, Shen H, Han L, Liu J, Xiao B, Li X, et al. A Multiparametric Fusion Radiomics Signature Based on Contrast-Enhanced MRI for Predicting Early Recurrence of Hepatocellular Carcinoma. J Oncol. (2022) 2022:3704987. doi: 10.1155/2022/3704987.
[3]Zhao Y, Wu J, Zhang Q, Hua Z, Qi W, Wang N, et al. Radiomics Analysis Based on Multiparametric MRI for Predicting Early Recurrence in Hepatocellular Carcinoma After Partial Hepatectomy. J Magn Reson Imaging. (2021) 53:1066-1079. doi: 10.1002/jmri.27424.
